# *Mangifera indica* ‘Namdokmai’ Prevents Neuronal Cells from Amyloid Peptide Toxicity and Inhibits BACE-1 Activities in a *Drosophila* Model of Alzheimer’s Amyloidosis

**DOI:** 10.3390/ph15050591

**Published:** 2022-05-11

**Authors:** Piya Temviriyanukul, Suwapat Kittibunchakul, Piyapat Trisonthi, Thanit Kunkeaw, Woorawee Inthachat, Dalad Siriwan, Uthaiwan Suttisansanee

**Affiliations:** 1 Food and Nutrition Academic and Research Cluster, Institute of Nutrition, Mahidol University, Salaya, Phuttamonthon, Nakhon Pathom 73170, Thailand; piya.tem@mahidol.ac.th (P.T.); suwapat.kit@mahidol.ac.th (S.K.); thanitmu7@yahoo.com (T.K.); woorawee.int@mahidol.ac.th (W.I.); 2 Institute of Food Research and Product Development, Kasetsart University, Bangkok 10900, Thailand; piyapat.tr@ku.th

**Keywords:** Alzheimer’s disease, cholinesterases, *Drosophila melanogaster*, enzyme inhibition, guava, mango, neuroprotective effect, phenolic profile, β-secretase

## Abstract

Alzheimer’s disease (AD) is a progressive neurological illness with few effective treatments. Thus, ameliorating the effects of AD using natural products has attracted global attention with promising efficacy and safety. In this study, ten tropical fruits including *Ananas comosus* ‘Phulae’, *Ananas comosus* ‘Pattavia’, *Carica papaya* ‘Khaekdum’, *Carica papaya* ‘Khaeknuan’, *Durio zibethinus* ‘Monthong’, *Durio zibethinus* ‘Chanee’, *Psidium guajava* ‘Kimju’, *Psidium guajava* ‘Keenok’, *Mangifera indica* ‘Kaew’ and *Mangifera indica* ‘Namdokmai’ were screened for their inhibitory activities against the key enzymes, cholinesterases and β-secretase (BACE-1), involved in AD pathogenesis. The top three fruit extracts with promising in vitro anti-AD activities were further investigated using rat pheochromocytoma PC-12 neuronal cell line and *Drosophila* AD model. Data showed that *M. indica* ‘Kaew’, *M. indica* ‘Namdokmai’ and *P. guajava* ‘Kimju’ reduced Aβ_1–42_-mediated neurotoxicity by promoting glutathione-dependent enzymes, while *M. indica* ‘Namdokmai’ limited Aβ_1–42_ peptide formation via BACE-1 inhibition and amended locomotory behavior of the *Drosophila* AD model. Results indicated the potential anti-AD properties of tropical fruits, particularly *M. indica* ‘Namdokmai’ in the prevention of Aβ_1–42_-mediated neurotoxicity and as a BACE-1 blocker.

## 1. Introduction

Alzheimer’s disease (AD) is the most general form of dementia, accounting for up to 75% of all cases and leading to death within 3 to 9 years after diagnosis [1,2]. AD pathogenesis is characterized by the aggregation of amyloid beta (Aβ) peptides into Aβ plaques deposited in the brain, resulting in neurotoxicity and apoptotic cell death [3]. Degradation of amyloid precursor protein (APP) to produce Aβ peptide composed mainly of 40 and 42 amino acid forms (Aβ_1–40_ or Aβ_1–42_) is proceeded via hydrolysis of β-secretase (BACE-1), followed by γ-secretase. Apparently, the difference in two amino acids of Aβ cause significantly different structures, bioactivities, and clinical behaviors. Aβ_1–42_ peptide is aggregated faster than Aβ_1–40_ peptide and becomes more neurotoxic. The cerebrospinal fluid Aβ_1–42/1–40_ ratio is currently used as an effective biomarker in AD pathology [4]. BACE-1 and Aβ aggregation inhibitors have been widely investigated as alternative pathways of AD treatments [5,6]. Other than the neurotoxicity of Aβ plaques, loss of cholinergic neurons via hydrolysis of the neurotransmitter, acetylcholine, by cholinesterases (ChEs), including acetylcholinesterase (AChE) and butyrylcholinesterase (BChE), can also lead to AD pathogenesis [7]. Several ChE inhibitors have been approved by the US Food and Drug Administration (FDA) as medicinal pathways to reduce AD symptomatic progression [8]. Additionally, brain neuronal cells are especially vulnerable to oxidative damage due to their high oxygen consumption rate and lack of antioxidant enzymes compared to other organs. Several studies have demonstrated that natural antioxidants could reduce or block neuronal apoptosis in AD pathophysiology [9].

Numerous plant phytochemicals have been reported to possess potential neuroprotective effects against AD by several mechanisms. The most common structurally diverse class of dietary phytochemicals is phenolics, previously reported as effective inhibitors of the key enzymes relevant to AD occurrence, while some act as Aβ aggregation inhibitors [6,10]. Gallic acid, a commonly found phenolic acid in most fruits, improved memory and spatial learning in AD-induced rats (via intrahippocampal injection of Aβ_1–42_) [11]. Flavonoids, as a sub-class of phenolics, are related to many functions in AD generation including antioxidant activity, reduction of β-amyloid aggregation, hyperphosphorylation of tau protein, gliosis and pro-inflammatory markers [12]. Flavonoids also elevate synaptic activity and pro-survival neurogenesis markers [13]. Various flavonoids such as apigenin, hesperetin, naringin, quercetin and rutin also improve the cognitive performance of animals in the Morris water maze [14]. 

Due to Thailand’s suitable climate (tropical wet and dry or savanna), the country has a large diversity of tropical fruits with vast potential biological activities [15]. Several tropical fruit extracts have been investigated to determine their inhibitory effects on key enzymes controlling obesity and diabetes as well as suppression of cancer initiation [15]. Extracts of Thai mulberry fruit cultivar ‘Chiang Mai’ have also been previously reported to exhibit anti-AD properties both in vitro and in vivo [16,17]. Interestingly, aqueous ethanolic extract of pineapple (*Ananas comosus*, Prima) has previously been reported to enhance cognitive function in scopolamine-induced mice [18], while a miniature review on the health benefits of papaya (*Carica papaya*, unknown variety) summarized its role in oxidative stress-induced AD [19]. A protective effect against H_2_O_2_-induced neurotoxicity of hot water extracted guava *(Psidium guajava*, unknown variety) has also been reported [20]. Additionally, ethanolic extract of durian (*Durio zibethinus*) cultivar ‘Monthong’ was able to inhibit neurotransmitter degrading acetylcholinesterase and Aβ aggregation and also had an enhanced neuroprotective effect against Aβ [21]. Aqueous ethanolic extract of mango (*Mangifera indica* ‘Namdokmai’) has also been reported to exhibit a neuroprotective effect and cognitive enhancement in mild cognitively impaired rats [22].

Despite this information, the anti-AD properties of some tropical fruits—especially Thai varieties—remain unavailable. Based on consumption popularity, tropical fruits including *A. comosus* ‘Phulae’, *A. comosus* ‘Pattavia’, *C. papaya* ‘Khaekdum’, *C. papaya* ‘Khaeknuan’, *D. zibethinus* ‘Monthong’, *D. zibethinus* ‘Chanee’, *P. guajava* ‘Kimju’, *P. guajava* ‘Keenok’, *M. indica* ‘Kaew’, and *M. indica* ‘Namdokmai’ were selected at their usual consumption maturity stages. Although anti-AD properties are available for some varieties of these fruits (*D. zibethinus* ‘Monthong’ and *M. indica* ‘Namdokmai’), it is interesting to compare the results with other varieties of the same species, with some having different consumption stages and others exhibiting different physical appearance, taste, and texture. The fruit extracts were screened for in vitro ChEs and BACE-1 inhibitory activities. The top three fruit extracts based on overall strong inhibitory activities were selected for further investigation of phenolic profiles by liquid chromatography–electrospray ionization–tandem mass spectrometry (LC-ESI-MS/MS), neurotoxicity, and neuroprotective effects using a neuronal-like PC-12 cell model and an in vivo *Drosophila* model of AD (co-expression of human APP and BACE-1). 

## 2. Results

### 2.1. In Vitro Screening on Enzyme Inhibition

Inhibition of the key enzymes relevant to AD including both ChEs (AChE and BChE) and BACE-1 of ten fruit extracts (*A*. *comosus* ‘Phulae’ and ‘Pattavia’, *C*. *papaya* ‘Khaekdum’ and ‘Khaeknuan’, *D*. *zibethinus* ‘Monthong’ and ‘Chanee’, *P*. *guajava* ‘Kimju’ and ‘Keenok’, and *M*. *indica* ‘Kaew’, and ‘Namdokmai’) were investigated to screen the top three extracts according to overall strong enzyme inhibitory activity for further analyses on phytochemical profiles, neurotoxicity, and neuroprotective effect on the PC-12 cells and *Drosophila* model of AD. Results indicated that *M*. *indica* and *P*. *guajava* with an extract concentration of 0.25 mg/mL exhibited higher AChE inhibitory activities (ranging 69–83% inhibitions) than the other fruit extracts (38–85% inhibitions) using higher extract concentration of 0.5 mg/mL (Table 1). Among these fruit extracts, *M*. *indica* ‘Namdokmai’ exhibited the highest AChE inhibitory activity followed by *P*. *guajava* ‘Keenok’, while *P*. *guajava* ‘Kimju’ and *M*. *indica* ‘Keaw’ equally held the third place.

Similar results were observed for BChE inhibitory activities (Table 1). *M*. *indica* and *P*. *guajava* at an extract concentration of 0.25 mg/mL exhibited higher BChE inhibitory activities (ranging 55–84% inhibitions) than the other fruit extracts (13–32% inhibitions) using a higher extract concentration of 0.5 mg/mL. Again, *M*. *indica* ‘Namdokmai’ exhibited the highest BChE inhibitory activity followed by both varieties of *P. guajava* and *M. indica* ‘Keaw’.

For BACE-1 inhibitory activities, *M*. *indica* and *P*. *guajava* exhibited higher inhibition (ranging 29–49% inhibitions) at extract concentration of 0.125 mg/mL than the other fruit extracts (27–59% inhibition) using higher extract concentration of 0.25 mg/mL (Table 1). Under the same extract concentration, *M*. *indica* ‘Namdokmai’ exhibited the highest BACE-1 inhibitory activity followed by *M*. *indica* ‘Keaw’ and *P*. *guajava* ‘Kimju’.

It is clear that *M*. *indica* ‘Namdokmai’ exhibited the highest inhibitory activities on AChE, BChE and BACE-1. Although *P*. *guajava* ‘Keenok’ exhibited the second highest AChE and BChE inhibitions, it came fourth for anti-BACE-1 inhibition. Therefore, *M*. *indica* ‘Keaw’ and *P*. *guajava* ‘Kimju’, with the second and third highest inhibitions on all three enzymes, were chosen. From the enzyme inhibitory screening results, *M*. *indica* ‘Namdokmai’, *M*. *indica* ‘Keaw’ and *P*. *guajava* ‘Kimju’ were selected for further analyses on phenolic profile and neuroprotective effect on PC-12 cells and *Drosophila* model of AD.

### 2.2. Phytochemical Profile

Utilizing 24 authentic standards of phenolics, phytochemicals of the selected fruit extracts with the top three highest enzyme inhibitions were investigated using LC-ESI-MS/MS analysis (Table 2, Supplementary Figures S1 and S2 and Supplementary Tables S1–S3). Only one phenolic acid, gallic acid, and one flavonoid, quercetin, were detected in both varieties of *M. indica*. Comparing between varieties, *M. indica* ‘Keaw’ exhibited 1.6 times higher gallic acid content than *M. indica* ‘Namdokmai’, while the latter exhibited 8.0 times higher quercetin content than the former. Likewise, *P. guajava* ‘Kimju’ contained only one phenolic acid, gallic acid; however, its content was 8.0–13.1 times lower than *M. indica*. *P. guajava* ‘Kimju’ contained four flavonoids including quercetin, isorhamnetin, naringenin and kaempferol, with contents of the last lower than the limit of detection (0.122 µg/mL) [23]. Interestingly, *P. guajava* ‘Kimju’ contained much higher quercetin than *M. indica* (up to 283.3 times higher). Lower contents of isorhamnetin and naringenin in *P. guajava* ‘Kimju’ were detected compared to quercetin.

### 2.3. Neurotoxicity of the Selected Fruit Extracts 

Toxicity of the selected fruit extracts (*M. indica* ‘Namdokmai’, *M. indica* ‘Kaew’ and *P. guajava* ‘Kimju’) was investigated using a resazurin assay to determine the neuronal-like PC-12 cells. The cells were incubated with the selected fruit extracts at different concentrations (25, 50, 100 and 150 µg/mL) for 24, 48 and 72 h before determining cell viability and compared to the untreated control (no fruit extracts). 

**Figure 1:**
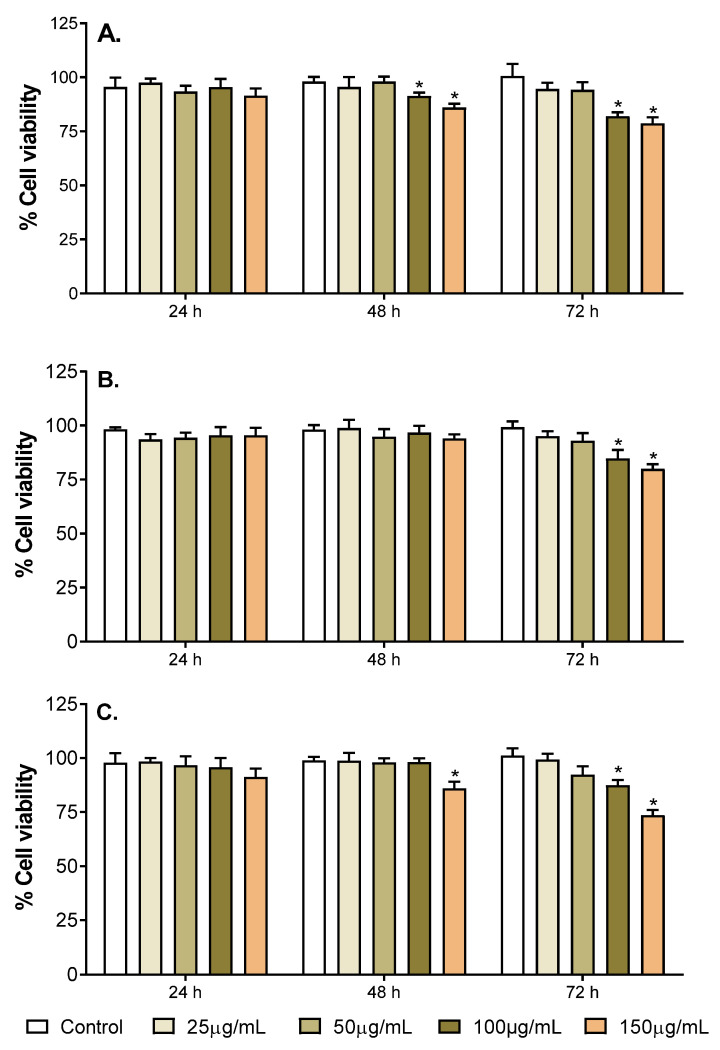
Percentage of cell viability when treating PC12 cells with different concentrations (25, 50, 100 and 150 µg/mL) of (**A**) *M. indica* ‘Kaew’, (**B**) *M. indica* ‘Namdokmai’, and (**C**) *P. guajava* ‘Kimju’ extracts at different incubating time periods (24, 48 and 72 h). Control: no exposure to fruit extracts (untreated control); * significance at *p* < 0.05 compared with untreated cells using two-way analysis of variance (ANOVA) followed by Tukey’s multiple comparisons test.

Results indicated that fruit extract concentrations did not cause cytotoxicity at 24–72 h time intervals (Figure 1 and Supplementary Table S4). At 24 h, no fruit extracts showed any sign of cytotoxicity on PC-12 cells at any concentrations. A significant reduction in cell viability in *M. indica* ‘Kaew’-treated cells was found when increasing incubating time to 48 and 72 h using extract concentrations of 100 and 150 µg/mL (Figure 1A). Interestingly, only 100 and 150 µg/mL of *M. indica* ‘Namdokmai’ extract significantly decreased cell viability at 72 h, while no significant difference in cell viability was observed at 48 h (Figure 1B). Similar to the effect of *M. indica* ‘Keaw’ extract, *P. guajava* ‘Kimju’ extract significantly reduced cell viabilities when increasing incubating time to 48 h using extract concentration of 150 µg/mL and 72 h using extract concentration of 100 and 150 µg/mL (Figure 1C). Nevertheless, all fruit extracts were determined to be not toxic to PC-12 cells, because more than 50% cell viabilities were achieved. 

### 2.4. Neuroprotective Effects of the Selected Fruit Extracts

Oxidative damage of PC-12 cells caused by either H_2_O_2_ or Aβ_1–42_ can induce apoptosis (cell death) in many biological and pathological pathways. In this study, neuroprotective effects of the selected fruit extracts (*M. indica* ‘Namdokmai’, *M. indica* ‘Kaew’ and *P. guajava* ‘Kimju’) on oxidative damage induced by H_2_O_2_ and Aβ_1–42_ were investigated. The PC-12 cells were treated with fruit extracts (25, 50, 100 and 150 µg/mL) for 24 h before treating with either H_2_O_2_ (200 µM) or Aβ_1–42_ (10 µM) for another 24 h. The viability of PC-12 cells treated with only oxidative inducers (inducer treated control) was then compared to the untreated control (no treatment of fruit extracts and oxidative inducers), while cell viability of PC-12 cells with exposure to both fruit extracts and oxidative inducers was compared to its inducer treated control (same oxidative inducer).

**Figure 2:**
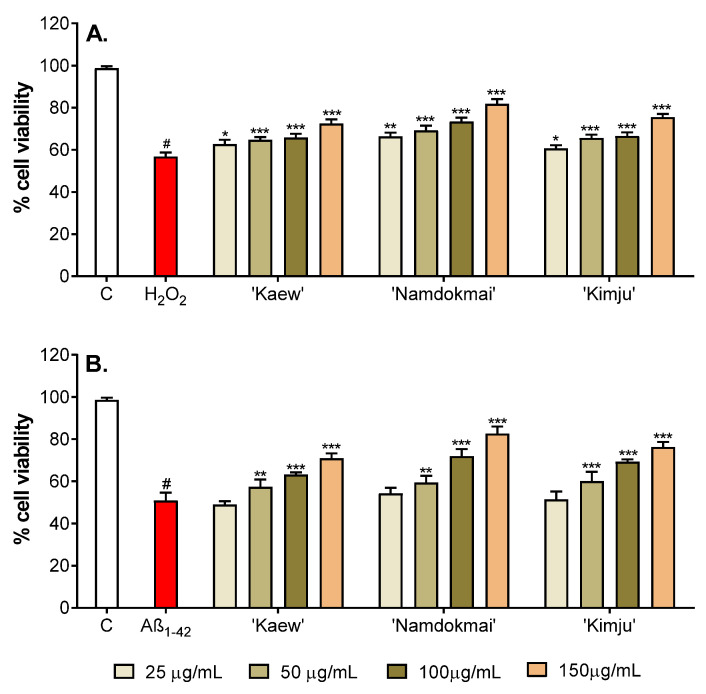
Neuroprotective effect of fruit extracts (*M. indica* ‘Kaew’, *M. indica* ‘Namdokmai’, and *P. guajava* ‘Kimju’) on oxidative damaged PC-12 cells examined using a resazurin assay. Percentage of cell viability induced by (**A**) H_2_O_2_ and (**B**) Aβ_1–42_ was compared to the untreated control group, while co-treatment between selected fruit extracts at different concentrations (25, 50, 100 and 150 µg/mL) and oxidative inducers were compared to its oxidative inducer treated group. C: untreated control; H_2_O_2_: H_2_O_2_-treated control; Aβ_1–42_: Aβ_1–42_-treated control; ^#^ significance at *p* < 0.05 compared to the untreated control, * significance at *p* < 0.05, ** significance at *p* < 0.01 and *** significance at *p* < 0.001 compared with either H_2_O_2_- or Aβ_1–42_-treated cells using one-way analysis of variance (ANOVA) followed by Tukey’s multiple comparisons test.

As shown in Figure 2A and Supplementary Table S5, treatment of PC-12 cells with H_2_O_2_ reduced cell viability to 56.82% compared to the untreated control. Incubation of PC-12 cells with 25–150 µg/mL of all selected fruit extracts significantly prevented the cytotoxic effects of H_2_O_2_. All fruit extracts significantly increased cell viability of PC-12 cells in a dose-dependent manner (60.68–81.90%). At the highest extract concentration (150 µg/mL), *M. indica* ‘Namdokmai’ showed a protective effect of more than 80%.

Similar to the cytotoxic effects of H_2_O_2_, Aβ_1–42_ induced cytotoxicity in PC-12 cells. After exposure to Aβ_1–42_, cell viability decreased to 50.92% (Figure 2B and Supplementary Table S5). However, no significant difference in cell viability was observed when treating PC-12 cells with the selected fruit extracts at the concentration of 25 µg/mL before Aβ_1–42_ exposure. Significant increase in cell viability was detected with fruit extracts at concentrations of ≥50 µg/mL compared to the Aβ_1–42_-treated group in a concentration-dependent manner. Again, *M. indica* ‘Namdokmai’ extract at concentration of 150 µg/mL increased cell viability to more than 80%.

Apoptotic cell death induced by oxidative damage also led to a loss of plasma membrane integrity, resulting in the release of cytosolic enzyme, i.e., lactate dehydrogenase (LDH). This stable enzyme is normally found in the cytosol of all cells; however, when the plasma membrane is damaged, it is rapidly released into the medium. In this study, the membrane protective effect of the selected fruit extracts was investigated. The LDH activity assay was used to measure membrane integrity in the form of cytoplasmic LDH quality released into the medium. If PC-12 cells induced with H_2_O_2_ caused an increase in LDH release compared to the untreated control, this implied that the integrity of the cell membrane was damaged. Similar to the cell viability measurement, PC-12 cells were treated with the selected fruit extracts (25, 50, 100 and 150 µg/mL) for 24 h before treating with oxidative inducers (200 µM H_2_O_2_ or 10 µM Aβ_1–42_) for another 24 h. Release of LDH was then determined using the LDH activity assay, and percentage of LDH released from PC-12 cells treated with only oxidative inducers (an inducer treated control) was determined compared to the untreated control (no treatment of both fruit extracts and oxidative inducers), while the percentage of LDH release of PC-12 cells with exposure to both fruit extracts and oxidative inducers was compared to its inducer treated control.

**Figure 3:**
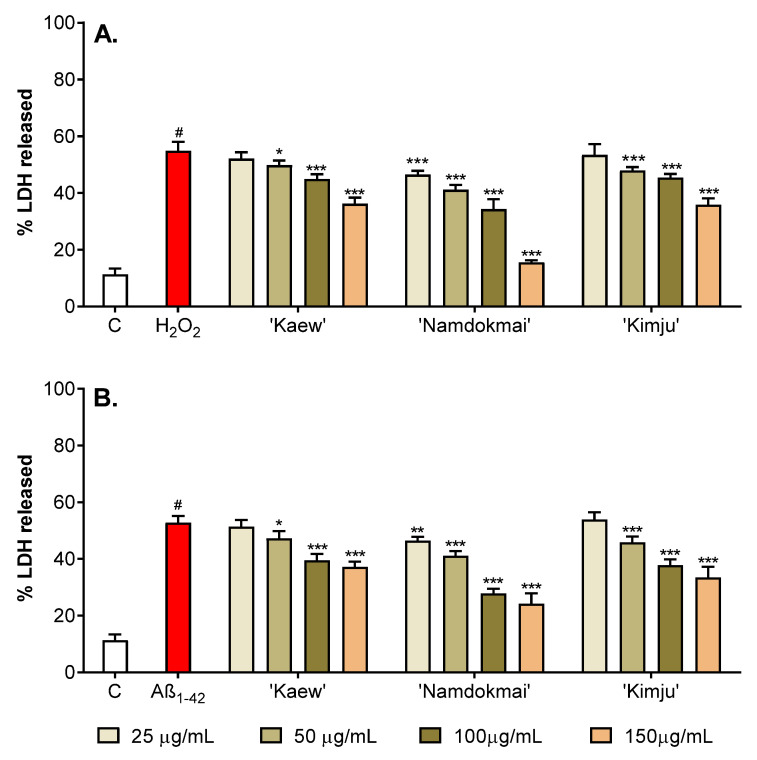
Neuroprotective effect of fruit extracts (*M. indica* ‘Kaew’, *M. indica* ‘Namdokmai’, and *P. guajava* ‘Kimju’) on oxidative damaged PC-12 cells examined using a lactate dehydrogenase (LDH) assay. Percentage of LDH released in PC-12 cells induced by (**A**) H_2_O_2_ and (**B**) Aβ_1–42_ was compared to the untreated control group, while co-treatment between selected fruit extracts at different concentrations (25, 50, 100 and 150 µg/mL) and oxidative inducers were compared to its oxidative inducer treated group. C: untreated control; H_2_O_2_: H_2_O_2_-treated control; Aβ_1–42_: Aβ_1–42_-treated control; ^#^ significance at *p* < 0.05 compared to the untreated control, * significance at *p* < 0.05, ** significance at *p* < 0.01 and *** significance at *p* < 0.001 compared with either H_2_O_2_- or Aβ_1–42_-treated cells using one-way analysis of variance (ANOVA) followed by Tukey’s multiple comparisons test.

As shown in Figure 3A and Supplementary Table S5, treating PC-12 cells with H_2_O_2_ increased LDH release to 54.83% compared to the untreated control, with LDH release of 13.10%. All selected fruit extracts significantly protected PC-12 cells from H_2_O_2_-induced oxidative stress. After exposure of PC-12 cells to various concentrations (25–150 µg/mL) of *P. guajava* ‘Kimju’ and *M. indica* ‘Kaew’ extracts, results indicated that release of LDH as a result of oxidative damage reduced with application of fruit extracts at concentration ≥50 µg/mL. By contrast, *M. indica* ‘Namdokmai’ extract effectively reduced LDH release at concentration ≥25 µg/mL. These results indicated that the selected fruit extracts, especially *M. indica* ‘Namdokmai’, protected cell apoptosis from H_2_O_2_-induced oxidative damage.

Cell toxicity caused by Aβ_1–42_-induced oxidative damage also led to LDH release. Results (Figure 3B and Supplementary Table S5) indicated that treating PC-12 cells with Aβ_1–42_ increased LDH release to 52.83%, compared to the untreated control with LDH release of 13.22%. Similar results on the neuroprotective effect of the fruit extracts against H_2_O_2_-induced oxidative damage were observed, in which *M. indica* ‘Namdokmai’ extract effectively reduced LDH release at concentration ≥25 µg/mL, while *P. guajava* ‘Kimju’ and *M. indica* ‘Kaew’ extracts reduced toxicity of Aβ_1–42_ at concentration ≥50 µg/mL.

Glutathione (GSH) is a predominant antioxidant inside mammalian cells and peripheral blood, with a significant function in oxidative damage protection of neuronal cells. Several studies have indicated an imbalance in GSH redox and altered GSH levels as AD pathology [24]. In this study, PC-12 cells were treated with oxidative inducers (200 µM H_2_O_2_ or 10 µM Aβ_1–42_) and fruit extracts at different concentrations (25, 50, 100 and 150 µg/mL) for 48 h before measuring GSH level to examine the neuroprotective effect of selected fruit extracts against oxidative damage. GSH levels of the oxidative inducer treated groups were compared with the untreated control, while the co-treatment groups (fruit extracts and oxidative inducers) were compared with their corresponding oxidative inducer treated groups.

**Figure 4:**
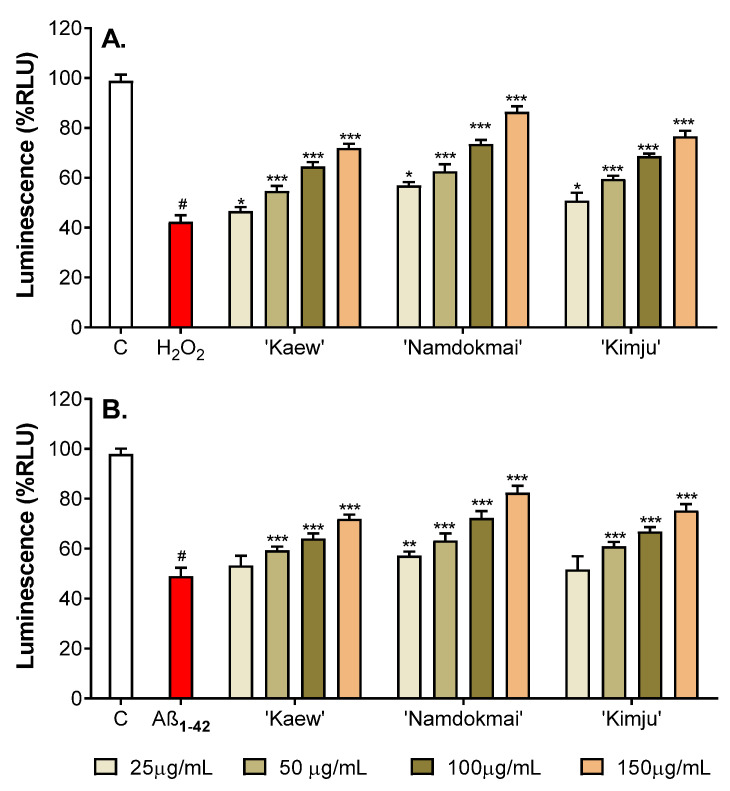
Neuroprotective effect of selected fruit extracts (*M. indica* ‘Namdokmai’, *M. indica* ‘Kaew’ and *P. guajava* ‘Kimju’) on oxidative stress in PC-12 cells investigated using a glutathione (GSH) assay. Antioxidant GSH level in PC-12 cells after treatment with (**A**) H_2_O_2_ and (**B**) Aβ_1–42_ was compared to the untreated control group. The GSH levels of the co-treatment between the selected fruit extracts at different concentrations (25, 50, 100 and 150 µg/mL) and oxidative inducers (H_2_O_2_ and Aβ_1–42_) were compared to its oxidative inducer treated group. RLU: relative light unit; C: untreated control; H_2_O_2_: H_2_O_2_-treated control; Aβ_1–42_: Aβ_1–42_-treated control; ^#^ significance at *p* < 0.05 compared to the untreated control, * significance at *p* < 0.05, ** significance at *p* < 0.01, and *** significance at *p* < 0.001 compared with oxidative inducer treated groups using one-way analysis of variance (ANOVA) followed by Tukey’s multiple comparisons test.

As shown in Figure 4A and Supplementary Table S5, the level of glutathione transferase (GST) in H_2_O_2_ treatment in PC-12 cells markedly decreased to 42.30%, compared to the untreated control group (98.92%). The level of GSH in H_2_O_2_-treated PC-12 cells was improved by the selected fruit extracts in a dose-dependent manner. The co-treatment of various concentrations of fruit extracts and H_2_O_2_ resulted in significant enhancement in GSH levels in the range of 46.63–86.47%. The highest GSH level was observed when PC-12 cells were treated with *M. indica* ‘Namdokmai’ extract, while the lowest was with *M. indica* ‘Kaew’.

Similar results were observed in PC-12 cells oxidatively damaged by Aβ_1–42_. As shown in Figure 4B and Supplementary Table S5, the Aβ_1–42_-treated group induced a decrease in GSH level to 49.00% compared to the untreated control group (97.90%). After co-treating cells with fruit extracts and Aβ_1–42_, GSH levels significantly increased, with the exception of *M. indica* ‘Kaew’ and *P. guajava* ‘Kimju’, at concentration of 25 µg/mL. The GSH levels increased in an extract concentration-dependent manner, especially *M. indica* ‘Namdokmai’ extract that protected GSH at almost the same level as the untreated control.

### 2.5. Anti-Alzheimer’s Disease Activities in Drosophila Model

**Figure 5:**
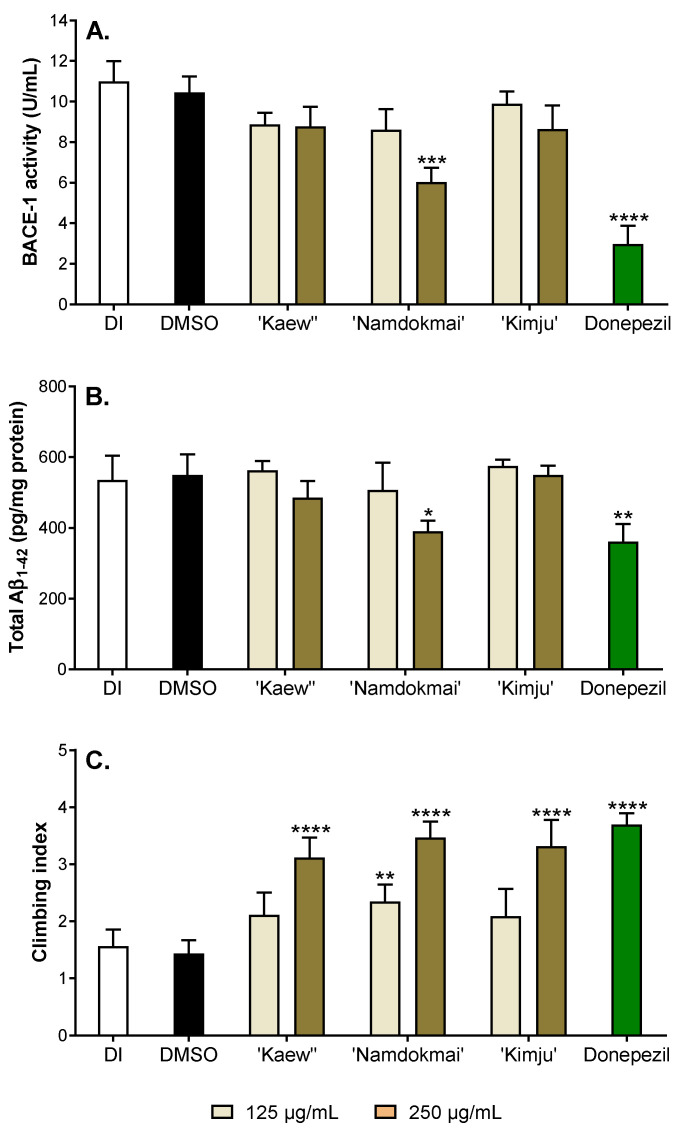
Effect of the fruit extracts (*M. indica* ‘Kaew’, *M. indica* ‘Namdokmai’, and *P. guajava* ‘Kimju’) on (**A**) β-secretase (BACE-1) activity, (**B**) total amount of amyloid beta 1–42 (Aβ_1–42_) level, and (**C**) fly climbing index. One- to two-day-old flies expressing human amyloid precursor proteins (APPs) and BACE-1 were treated with deionized water (DI), dimethyl sulfoxide (DMSO, solvent control) and selected fruits extracts (125 or 250 µg/mL) for 30 days before assayed for BACE-1, Aβ_1–42_ level or climbing index. * Significance at *p* < 0.05, ** significance at *p* < 0.01, and *** significance at *p* < 0.001 and **** significance at *p* < 0.0001 compared with a solvent control using one-way analysis of variance (ANOVA) followed by Tukey’s multiple comparisons test.

To provide in vivo supporting evidence for the anti-AD properties of the three selected fruit extracts (*M. indica* ‘Namdokmai’, *M. indica* ‘Kaew’ and *P. guajava* ‘Kimju’), the *Drosophila* model of AD was employed. The flies were co-expressed with both human APPs and BACE-1, specifically in the central nervous system. These flies eventually produced amyloid peptides, which is one of the hallmarks of AD; thereby representing the amyloid production pathway in AD cases. BACE-1 is a rate-limiting enzyme in the amyloid production pathway. Inhibition of BACE-1 results in reduced amyloid peptide levels, rendering it an important target for AD drugs. Therefore, the role of the fruit extracts on BACE-1 function in vivo was first explored as BACE-1 activity, Aβ_1–42_ formation and climbing index (Figure 5).

The 1–2-day-old AD flies were treated with safe doses (data not shown) of fruit extracts at 125 and 250 µg/mL for 30 days. Donepezil, a cholinesterase and BACE-1 inhibitor, was utilized as an AD drug control and 0.05% (*v*/*v*) dimethyl sulfoxide (DMSO) was used as solvent control. After 30 days of treatment, fly heads were collected and subjected to BACE-1 measurement. Flies that received DMSO exhibited high BACE-1 activity (10.45 U/mL), the same as flies that received deionized water (DI) (10.98 U/mL), while BACE-1 activity greatly reduced by 3.50 folds compared to DMSO-exposed flies, suggesting potential BACE-1 inhibition of donepezil, as previously demonstrated (Figure 5A). Flies treated with *M. indica* ‘Kaew’ and *P. guajava* ‘Kimju’ extracts, even at the highest dose similar to *M. indica* ‘Namdokmai’ at 125 µg/mL, exhibited a small reduction of BACE-1 activity that was not statistically significant. Interestingly, *M. indica* ‘Namdokmai’ extract at 250 µg/mL meaningfully reduced BACE-1 activity in the brain of AD flies (6.03 U/mL).

As previously mentioned, BACE-1 is involved in amyloid peptide production. The number of amyloid peptides was further quantified, especially the most cytotoxic form (Aβ_1–42_), in fly brains. Figure 5B illustrates that flies receiving DI or DMSO also showed high Aβ_1–42_ compared to flies treated with donepezil, implying that BACE-1 inhibition led to decreased Aβ_1–42_. As expected, *M. indica* ‘Kaew’, *P. guajava* ‘Kimju’ extracts and *M. indica* ‘Namdokmai’ at 125 µg/mL did not reduce amyloid peptide formation, while *M. indica* ‘Namdokmai’ extract at 250 µg/mL showed a clear reduction of Aβ_1–42_, confirming its role as a BACE-1 blocker in vivo.

Toxic amyloid peptides lead to poor locomotor ability of flies. Thus, to observe the toxic effect of Aβ_1–42_ (Figure 5B) and the rescuing effect of *M. indica* ‘Namdokmai’ extract at 250 µg/mL, the flies were subjected to locomotor ability assay at day 30. Flies expressing high levels of BACE-1 and Aβ_1–42_ (DI and DMSO) exhibited low climbing index, while donepezil-treated flies showed the highest climbing capabilities (Figure 5C). Low doses of *M. indica* ‘Kaew’ and *P. guajava* ‘Kimju’ extracts did not rescue the climbing index, in accordance with their high BACE-1 activities and Aβ_1–42_ contents, similar to DI and DMSO. Intriguingly, high doses of *M. indica* ‘Kaew’ and *P. guajava* ‘Kimju’ extracts as well as low and high doses of *M. indica* ‘Namdokmai’ extracts rescued the climbing capabilities of AD flies in the similar manner, suggesting that *M. indica* ‘Kaew’ and *P. guajava* ‘Kimju’ exerted their anti-AD properties, albeit not related to the amyloid cascade hypothesis.

## 3. Discussion

Several tropical fruits have been reported to possess biological activities with potential pharmaceutical applications in AD, a neurodegenerative disorder with no current treatment able to reverse brain cell impairment and cognitive loss. Thailand is one of the world’s largest tropical fruit suppliers, yet limited information on the anti-AD properties of Thai fruit varieties is available. In this study, ten fruit extracts including *A. comosus* ‘Phulae’ and ‘Pattavia’, *C. papaya* ‘Khaekdum’ and ‘Khaeknuan’, *D. zibethinus* ‘Monthong’ and ‘Chanee’, *P. guajava* ‘Kimju’ and ‘Keenok’ and *M. indica* ‘Kaew’ and ‘Namdokmai’ were investigated for their in vitro anti-AD properties through inhibition of the key enzymes relevant to AD including AChE, BChE and BACE-1. Results indicated that the top three fruit extracts with overall strong enzyme inhibitions were mango (*M. indica* ‘Namdokmai’ and *M. indica* ‘Keaw’) and guava (*P. guajava* ‘Kimju’). These three fruit extracts were further investigated regarding their phytochemical profiles as well as their neurotoxicity and neuroprotective effects using the neuronal-like PC-12 cell model and the *Drosophila* model of AD. Results suggested that the three fruit extracts exhibited various phenolics with potential AD key enzyme inhibitory properties and neuroprotective effects from H_2_O_2_- and Aβ_1–42_-induced oxidative damage. However, only *M. indica* ‘Namdokmai’ exhibited anti-AD properties by targeting the amyloid pathway in the fly model of Alzheimer’s amyloidosis.

Although not chosen for further investigation using cell culture and in vivo experiments, pineapple, papaya, and durian were previously reported for their anti-AD properties. Previous research indicated that pineapple (Prima) could improve cognitive deficit and memory performance in scopolamine-induced mice and suggested that pineapple flavonoids might be responsible for such activities due to previous research on anti-AChE activities of such flavonoids [18]. Temviriyanukul et al. (2021) reported that ferulic acid was the major phenolic detected in *A. comosus* ‘Pattavia’, while caffeic acid and hesperidin were predominant phenolics in *A. comosus* ‘Phulae’ [15]. Singh et al. (2021) summarized critical pathogenic factors of ferulic acid as well as its analogues and hybrids on the onset of AD [25], while a review on hesperidin as neuroprotective agent was also available [26]. Additionally, caffeic acid has previously been reported to exhibit AChE and BChE inhibitions with IC_50_ values of 4.21 and 5.60 µg/mL, respectively [27]. Despite no previous reports on the anti-AD properties of papaya, fermented papaya preparation was previously investigated for its neuroprotective role against copper-induced neurotoxicity [28]. Additionally, sinapic acid, a predominant phenolic detected in *C. papaya* ‘Khaekdum’ [15], expressed a neuroprotective effect on a Aβ_1–42_ induced AD mouse [29]. A protective effect against H_2_O_2_-induced neurotoxicity was also reported in guava [20]. Gallic acid, a predominant phenolic in *P. guajava* ‘Kimju’, and quercetin in *P. guajava* ‘Keenok’ were able to inhibit ChEs [30,31].

Chosen as an overall high anti-AD source in the present study, different parts of mango (*M. indica*) have been widely investigated regarding their phytochemical profiles as well as ChEs and BACE-1 inhibitions [32], but limited studies have been conducted on mango pulp. Previous studies suggested that the ripening stage enhanced phenolic contents of ‘Ataulfo’ mango from Mexico, while gallic acid as the most abundant phenolic decreased when the fruit became mature [33]. This result concurred with our results, indicating gallic acid as the major phenolic in both varieties of mango. *M. indica* ‘Keaw’, collected at a younger stage than *M. indica* ‘Namdokmai’, possessed higher gallic acid content. Other phenolic acids including chlorogenic acid, vanillic acid, and protocatechuic acid were also identified in lower amounts in this variety of mango [33]. *M. indica* ‘Haden’ from Brazil when completely ripe was reported to contain quercetin-3-*O*-glucoside [34]. This result also agreed with our findings of quercetin in both varieties of mango. Gallic acid and quercetin were also reported to exhibit ChEs inhibitory activities [30,31]. Interestingly, both phenolics inhibited BACE-1 activity as well as disrupted Aβ_1–42_ aggregation [35,36,37]. Thus, AChEs and BACE-1 inhibitory activities in mango might be a function of its predominant phenolic contents. Despite our hypothesis, only in vitro inhibitory activities on the enzymes relevant to AD in leaves and stem bark of mango are available [38,39], while no information on mango pulp has been reported.

Similar to mango, limited information exists on fruit pulp of guava compared to other parts, especially leaves, which have been widely studied [32,40]. Guava from Brazil extracted by acidic methanol was found to contain gallic acid, chlorogenic acid, ellagic acid, kaempferol, catechin, quercetin, and rutin [41]. Rutin, a quercetin glycoside or quercetin-3-*O*-rutinoside, was reported to be a major phenolics in guava [41], corresponding to our results that quercetin is the most abundant phenolic detected in *P. guajava* ‘Kimju’. Essential oil extracted from a Thai guava (unknown variety) indicated a 25% inhibition against AChE using fruit concentration of 0.1 mg/mL [42]. However, no previous information on in vitro BACE-1 inhibitory activity of guava pulp is available, making the present study the first report on the inhibitory activity of guava.

The three fruit extracts were further tested for their anti-AD properties in PC-12 neuronal cells. Figure 2 and Figure 3 show that all three fruit extracts prevented either H_2_O_2_ or Aβ_1–42_-mediated cytotoxicity in a dose-dependent manner. Hydrogen peroxide is well known as a strong oxidative agent, implying antioxidant properties of *M. indica* ‘Keaw’, *M. indica* ‘Namdokmai’, and *P. guajava* ‘Kimju’ previously reported in vitro [15]. This led the researchers to believe that Aβ_1–42_ peptides may cause high oxidative stress as of H_2_O_2_. Amyloid peptides trigger neuronal cell death via oxidative stress in PC-12 cells as H_2_O_2_ [43,44]. All three fruit extracts were rich in phytochemicals, particularly gallic acid and quercetin (Table 2). Hong et al. (2012) reported that gallic acid isolated from *Corni fructus* decreased Aβ_25–35_-induced intracellular reactive oxygen species (ROS) accumulation, apoptotic cells and caspase-3 activation in a dose-dependent manner in PC-12 cells (0.5–5.0 µM) [45], while Yu et al. (2020) showed that quercetin at 80 µM exhibited neuroprotectivity against amyloid peptides in PC-12 cells by induction of antioxidant enzymes including catalase (CAT), superoxide dismutase (SOD) and plasma glutathione peroxidase (GSH-Px). The same study also showed that quercetin significantly reduced malondialdehyde (MDA), a lipid peroxidation marker, in PC-12-cells exposed to amyloid peptides [46]. Below a concentration of 80 µM, quercetin lacked neuroprotective roles in these cells [46]. Our results (Figure 4) supported that treatment with the three fruit extracts rescued GSH levels in PC-12 cells exposed to Aβ_1–42_. GSH is a small peptide that contributes to the regulation of oxidative stress and redox balance [47] and is a crucial co-factor for antioxidant enzymes such as glutathione transferase (GST) and GSH-Px [48,49]. Thus, results in Figure 4 implied that fruit extracts restored a typical redox balance in PC-12 cells. In our study, the three crude fruit extracts at 150 µg/mL contained gallic acid and quercetin as *M. indica* ‘Keaw’ (6.90 and 0.01 µM), *M. indica* ‘Namdokmai’ (4.22 and 0.03 µM) and *P. guajava* ‘Kimju’ (0.53 and 0.10 µM). In mango, gallic acid may act as a bioactive agent quenching oxidative stress in PC-12 cells treated with amyloid peptides; meanwhile, in guava, other bioactive compounds may play dominant roles over quercetin. In addition to the anti-AD potential conferred by the antioxidant qualities of the three fruit extracts, reduction of aggregated amyloid peptides may be another mechanism to rescue PC-12 cell viability after amyloid peptide treatment. This notion is supported since gallic acid at 2.5 and 5.0 µM reduced Aβ_1–42_ aggregation, while it increased primary cortical neuron viability [35], similar to both *M. indica* ‘Keaw’ and *M. indica* ‘Namdokmai. Quercetin at levels from 1.0 µM prevented aggregation of amyloid peptides [37]. The quercetin content in our extracts was lower than in previous findings by 10–100 folds, implying the roles of unidentified bioactive compounds or their synergistic effect in guava.

To confirm the findings from both enzyme and cell studies (Figure 2 and Figure 3 and Table 1), the anti-AD properties of *M. indica* ‘Keaw’, *M. indica* ‘Namdokmai’ and *P. guajava* ‘Kimju’ were tested in the *Drosophila* model of amyloidogenic pathway. This *Drosophila* model has shown potential for anti-AD agent screening in several studies [50,51,52]. Advantages of AD flies are: (i) the UAS/GAL4 system, which enables targeted protein expression exclusively at desired tissues [53]; and (ii) the fly brain shows an open blood vascular system, thereby mimicking the human blood–brain barrier (BBB) known for its strong restriction to many biomolecules and medicines [54]. As a result, studies utilizing neuronal cell models rule out the significance of the BBB. The data in Figure 5 shows that only *M. indica* ‘Namdokmai’ exhibited anti-AD properties through inhibition of BACE-1, eventually resulting in reduced Aβ_1–42_ levels and improved locomotor ability of AD flies. Hence, this implies that bioactive agents in *M. indica* ‘Namdokmai’ extract or derivatives passed to the fly brain and impeded BACE-1 functions. The previous data demonstrated that gallic acid at 10 µM inhibited BACE-1 by approximately 20% [55], while quercetin at 5.4 µM inhibited BACE-1 by 50% [36]. At the highest dose in our *Drosophila* study, the concentrations of gallic acid and quercetin in *M. indica* ‘Keaw’, *M. indica* ‘Namdokmai’ and *P. guajava* ‘Kimju’ were (11.47, 0.01 µM), (7.04, 0.04 µM) and (0.88, 1.54 µM), respectively. Hence, along with the previously mentioned BACE-1 activity of gallic acid and quercetin, *M. indica* ‘Namdokmai’ exhibited significant anti-AD properties compared to *M. indica* ‘Keaw’, which was high in gallic acid, and *P. guajava* ‘Kimju’, which was high in quercetin (Table 2). Wattanathorn et al. (2014) also reported that the crude extract of *M. indica* ‘Namdokmai’ exhibited neuroprotective properties by improving memory impairment in rats with mild cognitive impairment (MCI), an intermediate state between normal cognition and dementia [22]. Other bioactive compounds may inhibit BACE-1 functions in fly brains. Mangiferin, a predominant natural compound occurring in *M. indica*, is suspected because this can cross the BBB [56] and exert anti-AD properties in transgenic AD mice [57]. However, mangiferin in mango flesh is quite low. This hypothesis requires further study because *M. indica* ‘Keaw’ lacked anti-AD potential in AD flies. In this study, only climbing ability, which is behavior assessment, was employed. Further experiments, such as analysis of fly neuroanatomy or cognitive functions are worthwhile.

The results obtained in this present study suggest potential anti-AD properties of these tropical fruits. The effective doses of the fruit extract with the strongest anti-AD properties as anti-AD agent in animal models should be further studied.

## 4. Materials and Methods

### 4.1. Sample Preparation and Extraction

Ten fruits, including *A. comosus* ‘Phulae’, *A. comosus* ‘Pattavia’, *C. papaya* ‘Khaekdum’, *C. papaya* ‘Khaeknuan’, *D. zibethinus* ‘Monthong’, *D. zibethinus* ‘Chanee’, *P. guajava* ‘Kimju’, *P. guajava* ‘Keenok’, *M. indica* ‘Kaew’, and *M. indica* ‘Namdokmai’, were purchased from vegetable and fruit market, Simummuang, in Lam Luk Ka District, Pathum Thani province, Thailand. Three fruits of the same variety were selected with the approximated weights as follows; 150–200 g for *A. comosus* ‘Phulae’, 2.5–3.0 kg for *A. comosus* ‘Pattavia’, 1.0–1.3 kg for *C. papaya* ‘Khaekdum’, 1.0–1.2 kg for *C. papaya* ‘Khaeknuan’, 2.5–3.0 kg for *D. zibethinus* ‘Monthong’, 2.6–3.0 kg for *D. zibethinus* ‘Chanee’, 250–300 g for *P. guajava* ‘Kimju’, 150–250 g for *P. guajava* ‘Keenok’, 200–300 g for *M. indica* ‘Kaew’, and 800–1000 g for *M. indica* ‘Namdokmai’. The main difference between *A. comosus* ‘Phulae’ and ‘Pattavia’ is that the former is smaller but has a sweeter taste than the latter. *C. papaya* ‘Khaekdum’ is normally consumed in its fully ripen stage as fresh fruit, while *C. papaya* ‘Khaeknuan’ is consumed in its younger stage as a salad. Similarly, *M. indica* ‘Namdokmai’ is consumed in its fully ripened stage, while *M. indica* ‘Kaew’ is normally consumed in a younger stage. *P. guajava* ‘Kimju’ and *P. guajava* ‘Keenok’ are consumed in their ripened stages. At this maturity stage, the color of *P. guajava* ‘Kimju’ flesh is white, while that of *P. guajava* ‘Keenok’ is pink. *D. zibethinus* ‘Monthong’ and *D. zibethinus* ‘Chanee’ are consumed at the same stage of ripening and have a unique aroma and texture. Identification and authentication of all samples and their ripening stages (as shown in Supplementary Table S6) were performed by Assoc. Prof. Dr. Chusri Trisonthi (Taxonomist, Faculty of Science, Chiang Mai University, Chiang Mai, Thailand) based on reliable references [58,59]. Sample preparation and extraction were performed according to a well-established protocol as previously reported [15]. Briefly, fresh samples were peeled, seeds removed (if any), cleaned, cut into smaller pieces, and dried using a −50 °C, 0.086 mbar freeze dryer (a Super Modulyo-230 from Thermo Fisher Scientific, Waltham, MA, USA) for 72 h. The dried samples were then ground using a grinder (a Philips 600W series from Philips Electronics Co., Ltd., Jakarta, Indonesia). Since three fruits of the same variety (total of 10 varieties) were selected and prepared as freeze-dried samples; thus, there were 30 dried samples to be extracted separately. The powdery samples (100 g) were extracted using a 2:2:1 ratio-solvent mixture of methanol, acetone, and water (400 mL) for 24 h. Solvent was removed by a rotary vacuum evaporator (Büchi Corporation, New Castle, DE, USA), and the crude extract was redissolved in distilled water (500 mL) before loading into a Sep-Pak C18 cartridge (Waters Corporation, Milford, MA, USA). Water-insoluble components were eluted from the column using ethyl acetate, and the solvent of the extract was removed using a rotary vacuum evaporator. The dried extracts were re-dissolved in 10% (*v*/*v*) DMSO before analysis.

### 4.2. Determination of Enzyme Inhibitory Activities

The inhibitory activities on the key enzymes relevant to AD were investigated using well-established protocols as previously reported [60], while the chemicals and reagents were purchased from Sigma-Aldrich (St. Louis, MO, USA). Briefly, the AChE and BChE assays were performed using enzymes, substrates, indicators, and extracts as indicated in Table 3. The fruit extracts were diluted with 10% (*v*/*v*) DMSO to the desired concentrations before forming the assays.

Enzyme kinetics was performed on a Synergy^TM^ HT 96-well UV–visible microplate reader and a Gen 5 data analysis software (BioTek Instruments, Inc., Winooski, VT, USA). The percentage of inhibition was calculated using the following equation:

(1) $$\mathrm{Percentage}\;(\%)\;\mathrm{of}\;\mathrm{inhibition}=100\times \left(1-\frac{B-b}{A-a}\right)$$

where *A* is the initial velocity (*v*_0_) of the reaction with enzyme but without the extract (control), *a* is the *v*_0_ of the reaction without enzyme and the extract (control blank), *B* is the *v*_0_ of the reaction with enzyme and the extract (sample), and *b* is the *v*_0_ of the reaction with the extract but without the enzyme (sample blank).

The BACE-1 reaction was an end-point assay, performed based on a fluorescence resonance energy transfer technique with an excitation wavelength (λ_ex_) at 320 nm and an emission wavelength (λ_em_) at 405 nm using a BACE-1 assay kit from Sigma-Aldrich (St. Louis, MO, USA). Percentage of inhibition was calculated using Equation (1), but instead of *v_0_*, the fluorescent absorbance at a particular wavelength was detected.

Donepezil was used as a standard reference in all three assays, and its IC_50_ values were calculated using a plot of percentage of inhibition versus donepezil concentration.

### 4.3. Determination of Phytochemical Profiles

The top three fruit extracts with the overall strong enzyme inhibitory activities were chosen to investigate their phytochemical profiles using LC–ESI-MS/MS. The conditions and validation of LC–ESI-MS/MS analysis in selective reaction monitoring (SRM) mode including retention time, linearity (linear range, linear regression, and correlation coefficient (R^2^)), limit of detection (LOD), limit of quantification (LOQ), percentage of relative standard deviation (%RSD), percentage of recovery (%recovery), fragment ions and radio frequencies (RF-lens) (Supplementary Tables S1 and S2) were followed a well-established procedure as previously reported [23]. The LC–ESI-MS/MS was performed on a Dionex Ultimate 3000 ultrahigh-performance liquid chromatography (UHPLC) system with a TSQ Quantis Triple Quadrupole mass spectrometer and a diode array detector (DAD) (Thermo Fisher Scientific, Bremen, Germany). The fruit extracts were loaded onto a 2.1 mm × 100 mm, 2.6 μm Accucore RP-MS column (Thermo Fisher Scientific, Bremen, Germany) with a mobile phase consisting of solvent A: acetonitrile and solvent B: 0.1% (*v*/*v*) aqueous formic acid and eluted using a 0.5 mL/min gradient elution at 10% A and 90% B for 10 min. A molecular mass analysis was performed on a Chromeleon 7 (version 7.2.9.11323) chromatography data system from Thermo Fisher Scientific (Bremen, Germany). A total of 24 authentic standards consisted of apigenin (>98.0% HPLC), caffeic acid (>98.0% HPLC), chlorogenic acid (>98.0% HPLC), *p*-coumaric acid (>98.0% gas chromatography (GC)), cinnamic acid (> 98.0% HPLC), 3,4-dihydroxybenzoic acid (≥97% Titration (T)), (−)-epigallocatechin gallate (>98.0% HPLC), ferulic acid (>98.0% GC), genistein (>98.0% HPLC), 4–hydroxybenzoic acid (>99.0% GC), hesperidin (> 90.0% HPLC), kaempferol (>97.0% HPLC), myricetin (>97.0% HPLC), luteolin (>98.0% HPLC), quercetin (>98.0% HPLC), naringenin (>93.0% HPLC), sinapic acid (>99.0% GC), and syringic acid (>97.0% T) from Tokyo Chemical Industry (Tokyo, Japan), rosmarinic acid (≥98% HPLC), gallic acid (97.5–102.5% T), rutin (≥94% HPLC), and vanillic acid (≥97% HPLC) from Sigma-Aldrich (St. Louis, MO, USA), isorhamnetin (≥99.0% HPLC) from Extrasynthese (Genay, France), and galangin (≥98.0% HPLC) from Wuhan ChemFaces Biochemical Co., Ltd. (Wuhan, China). The LC–ESI-MS/MS chromatogram of 24 authentic standards is shown in Supplementary Figure S1, while the chromatograms of the fruit samples are shown in Supplementary Figure S2.

### 4.4. Cell Culture Preparation

Rat Pheochromocytoma PC-12 cell line was purchased from American Type Culture Collection (ATCC) (Manassas, VA, USA). Cells were cultured in RPMI-1640 medium containing 10% (*v*/*v*) horse serum (HS), 5% (*v*/*v*) fetal bovine serum (FBS), 1% (*w*/*v*) L-glutamine, and 1% (*v*/*v*) Antibiotic-Antimycotic solution. The cells were grown at 37 °C in a 95% (*v*/*v*) humidified incubator with 5% (*v*/*v*) CO_2_. All cells were cultured in culture flasks precoated with poly-D-lysine. Exponentially growing cells in the final concentration of < 0.1% (*v*/*v*) DMSO were used for all assays. All chemicals and reagents were purchased from Thermo Fisher Scientific (Waltham, MA, USA).

### 4.5. Preparation of Aβ Peptides

An aliquot of 1 mM Aβ_1–42_ peptide (Bachem, Bubendorf, Switzerland) was prepared in hexafluoroisopropanol (HFIP), which was later removed by drying under N_2_ evaporation. The dry residue was then dissolved in phosphate buffer saline (PBS) to a final concentration of 100 μM and immediately frozen at −75 °C until required.

### 4.6. Cytotoxicity of the Selected Fruit Exacts

The metabolic activity of the PC-12 cells was determined using the CellTiter-Blue^®®^ Cell Viability Assay (Promega, Madison, WI, USA). To prepare seeded PC-12 cells, the PC-12 cells at a density of 1.0 × 10^4^ cells/well in a poly-D-lysine (PDL)-coated 96-well plate were incubated for 24 h at 37 °C using a 95% (*v*/*v*) humidified incubator with 5% (*v*/*v*) CO_2_. The seeded cells were then treated with the selected fruit extracts at different concentrations (25, 50, 100, and 150 µg/mL in 0.1% (*v*/*v*) DMSO) for 24, 48, and 72 h. At the end of the incubation time, 20 μL of resazurin dye was added to each well with additional incubation for 1 h at 37 °C. Fluorescence intensity at an excitation wavelength of 560 nm and an emission wavelength of 590 nm was measured using a 96-well microplate reader (Tecan infinite 200, Tecan Group Ltd., canton of Zürich, Switzerland). Cells treated with DMSO were used as a negative untreated control. Cell viability was present as a relative percentage of the untreated control.

### 4.7. Neuroprotective Effects against H_2_O_2_- and Aβ_1–42_-Induced Cytotoxicity

#### 4.7.1. Cell Viability Assay

The seeded PC-12 cells prepared as mentioned above were pretreated with the selected fruit extracts (0, 25, 50, 100, and 150 µg/mL in 0.1% (*v*/*v*) DMSO) for 24 h before adding 200 µM of H_2_O_2_ (Merck, Darmstadt, Germany) or 10 µM of Aβ_1–42_ and incubating for another 24 h. Cell survival as a relative percentage of cell viability was measured using resazurin dye as indicated above.

#### 4.7.2. Lactate Dehydrogenase (LDH) Assay

The LDH assay was performed using a Cytotoxicity LDH Assay Kit-WST (Dojindo Molecular Technologies, Inc., Kumamoto, Japan). The seeded PC-12 cells were pretreated with the selected fruits extracts (0, 25, 50, 100, and 150 µg/mL in 0.1% (*v*/*v*) DMSO) for 24 h and treated with 200 µM H_2_O_2_ or 10 µM Aβ_1–42_ for another 24 h. The measurement of LDH activities followed the manufacturer’s instruction, and the results were expressed as percentage of LDH released.

#### 4.7.3. Glutathione (GSH) Assay

Intracellular GSH was evaluated using the GSH-Glo^TM^ Glutathione assay (Promega, Madison, WI, USA). The seeded PC-12 cells were treated with 200 µM H_2_O_2_ or 10 µM Aβ_1–42_ and the selected fruit extracts (0, 25, 50, 100, and 150 µg/mL in 0.1% (*v*/*v*) DMSO) for 48 h. The GSH assay was performed following the manufacturer’s instruction, and the results were expressed as percentage of relative light unit (RLU) of luminescence.

### 4.8. Drosophila Stock, Culture and Treatment

Flies (elav-GAL4, BDSC 8760 and UAS-APP-BACE-1, BDSC 33798) were received from the Bloomington Stock Center (BDSC) at Indiana University. Mating between elav-GAL4 and UAS-APP-BACE-1 resulted in the F1 progeny flies expressing human APP and BACE-1 in the central nervous system. The flies were administrated via their food consumption. In brief, groups of 100 newly eclosed F1 flies were cultured on Formula 4–24 blue^®®^ medium (Carolina, Burlington, NC, USA) supplemented with safety doses of *M. indica* ‘Keaw’, *M. indica* ‘Namdokmai’ or *P. guajava* ‘Kimju’ (125 or 250 µg/mL), 10 µM donepezil (drug control), DI (negative control), or 0.05% (*v*/*v*) DMSO (solvent control) at 28 °C for 30 days. To keep the food fresh, it was replenished every three days.

### 4.9. Drosophila Locomotor Assay

After 30 days of treatment, flies in each treatment were separated into three groups and put into a clear tube without anesthetic. They were then allowed to rest at room temperature for 20 min. The tube was then tapped to bring all flies to the bottom of the tube. Following tapping, the climbing rate was collected and examined as described earlier [61]. Three experiments were conducted independently.

### 4.10. Determination of Aβ_1–42_ Peptide by Enzyme-Linked Immunosorbent Assay (ELISA) and BACE-1 Activity in Drosophila Brain

Quantification of Aβ peptide and BACE-1 activity in fly brain were conducted as formerly detailed without modification [50]. In brief, for quantification of Aβ_1–42_ peptide, 25 to 30 fly heads were homogenized in a 5 M guanidine-HCl containing protease Inhibitor Cocktail (Thermo Fisher Scientific, Waltham, MA, USA) and centrifuged at 12,000× *g* for 15 min at 4 °C. The supernatant was then subjected to human Aβ42 ELISA kit (Life Technologies, Invitrogen, Carlsbad, CA, USA). Concentration of Aβ was determined by comparing with standard Aβ_1–42_. For BACE-1 activity, the same numbers of fly heads were homogenized in T-PER™ Tissue Protein Extraction Reagent (Thermo Fisher Scientific, Waltham, MA, USA) without protease inhibitor. The lysate was then subjected to BACE-1 activity detection kit (Sigma-Aldrich, St. Louis, MO, USA). One unit of BACE-1 activity means it hydrolyzes 1.0 picomole of 7-Methoxycoumarin-4-acetyl-[Asn^670^, Leu^671^]- Amyloid β/A4 Precursor Protein 770 Fragment 667-676- (2,4-dinitrophenyl)Lys-Arg-Arg amide substrate per minute at pH 4.5 at 37 °C.

### 4.11. Statistical Analysis

All experiments were performed in triplicate (*n* = 3) or as indicated otherwise. The results are presented as mean ± standard deviation (SD). One–way or two-way analysis of variance (ANOVA) with Duncan’s multiple comparison test or Tukey’s multiple comparisons test (more than two data) were used to state significant difference among values. For the comparison of two data sets, significant differences between values using Student’s unpaired *t*-test were employed. Statistically analyses were performed using the statistical package for the social sciences (version 18 for Windows, SPSS Inc., Chicago, IL, USA).

## 5. Conclusions

The in vitro results indicated the overall strong AChE, BChE, and BACE-1 inhibitory activities of mango (*M. indica* ‘Namdokmai’ and *M. indica* ‘Keaw’) and guava (*P. guajava* ‘Kimju’) extracts, suggesting their potential roles in anti-AD properties. These three extracts were thus further investigated using cell culture and *Drosophila* model of AD. Additionally, we hypothesized that phenolics might be responsible for the in vitro activities. However, it is difficult to relate phenolics to their bioactivities due to limited information on LC-ESI-MS/MS analysis, which only presents the phenolic profiles of the selected fruit extracts (*M. indica* ‘Namdokmai’, *M. indica* ‘Keaw’ and *P. guajava* ‘Kimju’) and only in vitro experiments may not be sufficient to define inactive extracts. Thus, excluding seven extracts could be considered a limitation of the present study. Nevertheless, *M. indica* ‘Namdokmai’ extract showed promising anti-AD potential by preventing Aβ-induced oxidative stress in neuronal cells and inhibiting BACE-1 in AD flies. Elucidation of the bioactive agents or effective doses in higher animal models, however, requires comprehensive further investigation.

## Figures and Tables

**Table 1:** Inhibitory activities on the key enzymes related to Alzheimer’s disease (AD) of ten fruit extracts in comparison to an AD drug, donepezil.

Fruit Extracts		Enzyme Inhibitory Activities (%Inhibition)		
		AChE ^1^	BChE ^1^	BACE-1 ^2^
*Ananas comosus*	‘Pattavia’	84.67 ± 1.99 ^a^	18.94 ± 0.97 ^d^	58.56 ± 4.26 ^a^
	‘Phulae’	55.81 ± 1.51 ^c^	24.48 ± 0.65 ^c^	39.29 ± 1.98 ^c^
*Carica papaya*	‘Khaekdum’	54.89 ± 0.36 ^c^	32.05 ± 1.46 ^a^	41.11 ± 3.31 ^c^
	‘Khaeknuan’	49.19 ± 0.80 ^d^	27.35 ± 2.09 ^b^	41.34 ± 1.02 ^c^
*Durio zibethinus*	‘Chanee’	58.68 ± 0.70 ^b^	21.39 ± 2.04 ^d^	51.58 ± 2.67 ^b^
	‘Monthong’	38.36 ± 0.25 ^e^	13.34 ± 0.51 ^e^	27.34 ± 1.50 ^d^
*Mangifera indica*	‘Namdokmai’	83.14 ± 0.4 ^A^	84.14 ± 0.64 ^A^	48.88 ± 2.57 ^A^
	‘Keaw’	69.12 ± 0.21 ^C^	54.72 ± 1.61 ^C^	36.89 ± 1.65 ^B^
*Psidium guajava*	‘Kimju’	69.69 ± 0.41 ^C^	70.31 ± 2.50 ^B^	32.09 ± 1.13 ^C^
	‘Keenok’	70.69 ± 0.83 ^B^	68.95 ± 0.55 ^B^	28.59 ± 0.90 ^D^
Donepezil (IC_50_) (µM)		3.12 ± 0.37	2.14 ± 0.43	1.31 ± 0.07

All data are expressed as mean ± standard deviation (SD) of triplicate experiments (*n* = 3). Different capital letters indicate significantly different enzyme inhibitions of *M. indica* and *P. guajava*, while different lowercase letters indicate significantly different enzyme inhibitions of *A. comosus*, *C. papaya*, and *D. zibethinus* in the same enzyme assay at *p* < 0.05 calculated by one-way analysis of variance (ANOVA) and Duncan’s multiple comparison test. AChE: acetylcholinesterase; BChE: butyrylcholinesterase; BACE-1: β-secretase; IC_50_: half maximal inhibitory concentration; ^1^ concentration of fruit extracts = 0.5 mg/mL, except for *M. indica* and *P. guajava*, which used 0.25 mg/mL; ^2^ concentration of fruit extracts = 0.25 mg/mL, except for *M. indica* and *P. guajava*, which used 0.125 mg/mL.

**Table 2:** Phenolic profiles of *M. indica* ‘Namdokmai’, *M. indica* ‘Kaew’, and *P. guajava* ‘Kimju’ extracts using liquid chromatography–electrospray ionization tandem mass spectrometry (LC–ESI-MS/MS).

Phenolics (µg/g Extract)	*Mangifera indica*		*Psidium guajava*
	‘Kaew’	‘Namdokmai’	‘Kimju’
Gallic acid	7802.07 ± 199.68 ^aA^	4790.75 ± 36.74 ^aB^	596.80 ± 12.98 ^bC^
Quercetin	6.59 ± 0.44 ^bB^	52.65 ± 5.23 ^bB^	1867.27 ± 138.86 ^aA^
Naringenin	ND	ND	3.50 ± 0.34 ^c^
Kaempferol	ND	ND	<LOD
Isorhamnetin	ND	ND	53.73 ± 2.27 ^c^

All data are expressed as the mean ± standard deviation (SD) of triplicate experiments (*n* = 3). Different lowercase letters indicate significantly different contents of phenolics in the same fruit extracts, while different capital letters indicate significantly different contents of the same phenolics in different fruit extracts at *p* < 0.05 using one-way analysis of variance (ANOVA) and Duncan’s multiple comparison test (more than two data) or Student’s unpaired *t*–test (two data). ND: not detected; LOD: limit of detection.

**Table 3:** Assay components for determination of acetylcholinesterase (AChE) and butyrylcholinesterase (BChE) inhibitory activities.

Assay	Enzyme (100 μL)	Substrate (40 μL)	Indicator (10 µL)	Extract (40 µL)	Detection Wavelength
AChE	20 ng *Electrophorus electricus* AChE (1000 units/mg)	0.8 mM acetylthiocholine	16 mM 5,5′-dithiobis(2-nitrobenzoic acid)	0.25–0.5 mg/mL	412 nm
BChE	50 ng equine serum BChE (≥10 units/mg)	0.4 mM butyrylthiocholine			

## Data Availability

Data are contained within this article and the Supplementary Materials.

## Supplementary Materials

The following are available online at https://www.mdpi.com/article/10.3390/ph15050591/s1, Figure S1: The liquid chromatography–electrospray ionization tandem mass spectrometry (LC–ESI-MS/MS) chromatograms of 24 phenolic standards, Figure S2: The liquid chromatography–electrospray ionization tandem mass spectrometry (LC–ESI-MS/MS) chromatograms of the selected fruit samples including (**a**) *Mangifera indica* ‘Kaew’, (**b**) *Mangifera indica* ‘Namdokmai’, and (**c**) *Psidium guajava* ‘Kimju’, Table S1: The validation parameters including retention time, linearity (linear range, linear regression, and correlation coefficient (R^2^)), limit of detection (LOD), limit of quantification (LOQ), percentage of relative standard deviation (%RSD), and percentage of recovery (%recovery) in selective reaction monitoring (SRM) mode of 24 authentic standards of phenolics using liquid chromatography−electrospray ionization−tandem mass spectrometry (LC-ESI-MS/MS), Table S2: Fragment ions (ion mass, parent ions, selective reaction monitoring (SRM) transition and collision energy) and radio frequencies (RF-lens) of 24 authentic standards of phenolics using liquid chromatography−electrospray ionization−tandem mass spectrometry (LC-ESI-MS/MS), Table S3: Parameters (mass spectral transition, retention time and area under the curve) for liquid chromatography–electrospray ionization tandem mass spectrometry (LC–ESI-MS/MS), Table S4: Information on neurotoxicity of the selected fruit extracts (*M. indica* ‘Namdokmai’, *M. indica* ‘Keaw’ and *P. guajava* ‘Kimju’), Table S5: Information on neuroprotective effect of the selected fruit extracts (*M. indica* ‘Namdokmai’, *M. indica* ‘Keaw’ and *P. guajava* ‘Kimju’), Table S6: Images of fruit samples including *Ananas comosus* ‘Phulae’, *Ananas comosus* ‘Pattavia’, *Carica papaya* ‘Khaekdum’, *Carica papaya* ‘Khaeknuan’, *Durio zibethinus* ‘Monthong’, *Durio zibethinus* ‘Chanee’, *Psidium guajava* ‘Kimju’, *Psidium guajava* ‘Keenok’, *Mangifera indica* ‘Kaew’, and *Mangifera indica* ‘Namdokmai’.
