# Supplementary material for: Mangifera indica ‘Namdokmai’ Prevents Neuronal Cells from Amyloid Peptide Toxicity and Inhibits BACE-1 Activities in a Drosophila Model of Alzheimer’s Amyloidosis
Source: Pharmaceuticals (Basel). 2022 May 11;15(5):591. doi: 10.3390/ph15050591 (PMC9146065; doi:10.3390/ph15050591)

Supplementary materials

Mangifera indica 'Namdokmai' prevents neuronal cells from amyloid peptide toxicity and inhibits BACE-1 activities in a *Drosophila* model of Alzheimer's amyloidosis

Piya Temviriyanukul [1], Suwapat Kittibunchakul [1], Piyapat Trisonthi [2], Thanit Kunkeaw [1], Woorawee Inthachat [1], Dalad Siriwan [2,*,†] and Uthaiwan Suttisansanee [1,*,†]

1 Food and Nutrition Academic and Research Cluster, Institute of Nutrition, Mahidol University, Salaya, Phuttamonthon, Nakhon Pathom 73170, Thailand; piya.tem@mahidol.ac.th (P.T.); suwapat.kit@mahidol.ac.th (S.K.); thanitmu7@yahoo.com (T.K.); woorawee.int@mahidol.ac.th (W.I.)
2 Institute of Food Research and Product Development, Kasetsart University, Chatuchak, Bangkok 10900, Thailand; piyapat.tr@ku.th (P.Tr); dalad.s@ku.th (D.S.)
* Correspondence: dalad.s@ku.th (D.S.); uthaiwan.sut@mahidol.ac.th (U.S.)
† These authors contributed equally to this work.

Supplementary Table S1:

The validation parameters including retention time, linearity (linear range, linear regression, and correlation coefficient (R^2)), limit of detection (LOD), limit of quantification (LOQ), percentage of relative standard deviation (%RSD), and percentage of recovery (%recovery) in selective reaction monitoring (SRM) mode of 24 authentic standards of phenolics using liquid chromatography–electrospray ionization–tandem mass spectrometry (LC-ESI-MS/MS).

Compounds	Standards	Retention time (min)	Linear range (µg/mL)	Linear regression equation	Correlation coefficient (R^2)	LOD (µg/mL)	LOQ (µg/mL)	%RSD (Inter-day)	%Recovery		
									Low level (µg/mL)	Medium level (µg/mL)	High level (µg/mL)
1	Epigallocatechin gallate	0.440	0.125–40	y =8533x + 1053.4	0.9985	0.067	0.230	0.023	91.84	85.36	91.37
2	Gallic acid	0.564	0.195–25	y =3323.1x – 2100.4	0.9984	0.04	0.14	0.01	113.05	118.57	109.12
3	3,4-Dihydroxybenzoic acid	0.803	0.195–25	y =11490x – 10877	0.9935	0.010	0.034	0.003	90.59	85.75	89.75
4	Chlorogenic acid	0.922	0.3125–40	y =8377.5x – 3623.5	0.9934	0.017	0.055	0.006	91.94	87.50	95.02
5	4-Hydroxybenic acid	1.160	0.3125–40	y =2482.6x– 3998.4	0.9917	0.027	0.090	0.009	109.67	103.60	101.28
6	Caffeic acid	1.400	0.3125–40	y =12328x–19725	0.9918	0.010	0.035	0.003	105.36	93.98	87.41
7	Syringic acid	1.539	3.125–100	y =68.091x + 230.43	0.9955	0.582	1.939	0.194	116.35	97.42	94.91
8	Vanillic acid	1.630	2.5–100	y =213.67x – 975.72	0.9900	0.15	0.48	0.05	99.86	101.76	100.12
9	*p*-Coumaric acid	2.452	0.3125–40	y =8532.4x–13559	0.9910	0.013	0.042	0.004	88.22	81.36	98.05
10	Rutin	2.737	0.009–1.25	y =49729x – 33.064	0.9999	0.001	0.005	0.0005	94.63	114.00	108.73
11	Sinapic acid	2.772	0.39–25	y =1592.6x – 832.22	0.9977	0.026	0.086	0.009	81.34	92.16	84.22
12	Ferulic acid	2.851	1.56–100	y =559.03x – 1819.2	0.9947	0.155	0.518	0.052	91.51	89.24	93.10
13	Hesperidin	3.410	0.25–40	y =838.63x – 242.2	0.9986	0.07	0.22	0.02	100.43	104.06	108.60
14	Myricetin	3.431	1.25–40	y =303.47x – 601.81	0.9976	0.261	0.871	0.087	113.07	81.77	91.12
15	Rosmarinic acid	3.528	0.3125–40	y =4322.4x-3744.1	0.9956	0.07	0.25	0.02	92.45	106.35	99.62
16	Luteolin	4.158	0.195–12.5	y =8381.9x – 5000.7	0.9945	0.015	0.050	0.0005	84.21	96.21	107.09
17	Quercetin	4.185	0.05–12.5	y =2934x + 917.17	0.9937	0.05	0.18	0.02	83.36	115.06	95.74
18	Cinnamic acid	4.522	0.039–10	y =6631.9x – 866.59	0.9964	0.049	0.163	0.016	101.94	98.84	95.85
19	Apigenin	4.689	0.34–11	y =1790.7x – 287.7	0.9997	0.127	0.424	0.042	88.84	106.89	114.79
20	Genistein	4.693	0.625–40	y =1247.2x – 1747.1	0.9977	0.049	0.163	0.016	95.33	101.49	11633
21	Naringenin	4.705	0.0008–5	y =16755x + 443.03	0.9932	0.003	0.011	0.001	117.92	96.26	111.08
22	Kaempferol	4.790	0.25–10	y =1006.8x – 346.28	0.9905	0.122	0.406	0.041	92.35	107.69	102.17
23	Isorhamnetin	4.878	0.0098–2.5	y =12698x + 586.16	0.9945	0.016	0.052	0.005	113.57	105.88	111.14
24	Galangin	6.146	0.3125–40	y =5012.1x – 9354.7	0.9879	0.010	0.035	0.003	84.01	112.92	115.80

Note: These data were from Sirichai et al., 2022 [23]. A linear relationship in the calibration plots of all standards presented a correlation coefficients (R^2), which was higher than 0.99. The detection limits to quantify phenolics were indicated as LOD and LOQ. The repeatability of peak areas within the same day was present as %RSD. The range of 80-120% recovery percentage was within acceptable limits. Thus, this LC–ESI-MS/MS analysis with SRM mode was suitable and reliable to quantify phenolics in the extracts.

Supplementary Table S2:

Fragment ions (ion mass, parent ions, selective reaction monitoring (SRM) transition and collision energy) and radio frequencies (RF-lens) of 24 authentic standards of phenolics using liquid chromatography–electrospray ionization–tandem mass spectrometry (LC-ESI-MS/MS).

**Compounds**	**Standards**	**Ion mass**	**Parent ions (*m*/*z*)**	**Selective reaction monitoring (SRM) transitions (*m*/*z*) and collision energy (*V*)**	**Radio frequencies (RF-lens) (*V*)**
1	Epigallocatechin gallate	[M–H]	457.175	305.155 (16.84 V), 168.97 (17.59 V), 125.042 (40.30 V)	204
2	Gallic acid	[M–H]	169.05	124.988 (14.56 V), 96.917 (18.77 V), 79.185 (22.94 V)	147
3	3,4-Dihydroxybenzoic acid	[M–H]	152.95	109.113 (14.35 V), 81.042 (20.50 V), 91.042 (24.59 V)	128
4	Chlorogenic acid	[M–H]	353.075	179.042 (14.06 V), 191.000 (16.54 V), 85.095 (39.96 V)	148
5	4-Hydroxybenic acid	[M–H]	137.05	92.970 (14.86 V), 65.000 (29.39 V), 75.000 (31.96 V)	110
6	Caffeic acid	[M–H]	179.038	135.054 (15.07 V), 107.071 (22.57 V), 85.042 (31.96 V)	151
7	Syringic acid	[M–H]	197.138	182.185 (13.72 V), 167.113 (19.24 V), 123.095 (22.31 V)	130
8	Vanillic acid	[M–H]	167.000	123.042 (11.66 V), 151.97 (14.59 V), 108.042 (18.65 V)	114
9	*p*-Coumaric acid	[M+H]	165.05	147.054 (11.70 V), 119.113 (19.36 V), 91.125 (25.89 V)	90
10	Rutin	[M+H]	611.20	303.13 (20.80), 465.20 (12.71V)	198
11	Sinapic acid	[M–H]	223.25	208.125 (13.51 V), 164.024 (15.78 V), 192.970 (22.65 V)	141
12	Ferulic acid	[M–H]	192.95	149.125 (11.28 V), 177.970 (13.05 V), 134.042 (16.50 V)	124
13	Hesperidin	[M–H]	609.30	301.179 (24.50 V), 325.179 (27.83 V), 286.125 (41.60 V)	299
14	Myricetin	[M–H]	317.088	178.970 (19.53 V), 150.988 (24.50 V), 137.113 (26.86 V)	245
15	Rosmarinic acid	[M–H]	359.20	197.000 (15.70 V), 161.113 (17.38 V), 133.054 (37.81 V)	175
16	Luteolin	[M–H]	285.138	197.000 (15.70 V), 161.113 (17.38 V), 133.054 (37.81 V)	241
17	Quercetin	[M–H]	301.200	178.976 (18.18 V), 273.125 (19.45 V), 151.042 (21.39 V)	237
18	Cinnamic acid	[M–H]	147.00	103.00 (11.23V), 77.083 (23.07)	107
19	Apigenin	[M–H]	269.075	116.863 (34.28 V), 149.071 (25.13 V), 151.131 (25.05 V)	244
20	Genistein	[M–H]	269.138	224.054 (25.60 V), 159.054 (29.26 V), 132.929 (30.95 V)	239
21	Naringenin	[M+H]	272.938	146.97 (21.01 V), 153.054 (24.42 V), 119.000 (31.28 V)	160
22	Kaempferol	[M–H]	285.150	184.911 (25.85 V), 239.113 (27.03 V), 186.988 (28.17 V)	260
23	Isorhamnetin	[M–H]	315.088	300.000 (21.30 V), 150.970 (29.14 V), 271.054 (30.57 V)	233
24	Galangin	[M+H]	271.088	165.042 (28.80 V), 197.125 (31.75 V), 153.113 (32.42 V)	248

Note: These data were from Sirichai et al., 2022 [23].

Supplementary Table S3:

Parameters (mass spectral transition, retention time and area under the curve) for liquid chromatography–electrospray ionization tandem mass spectrometry (LC–ESI-MS/MS) of fruit samples.

Fruits	Standard	MS transition		Retention time (min)	Area under the curve
		Transition 1	Transition 2		
Mangifera indica 'Kaew'	Gallic acid	169.05 / 124.99	169.05 / 79.18	0.546-0.556	158046.00-151473.00
	Quercetin	301.20 / 151.04	301.20 / 178.98	4.135-4.151	907.00-1087.00
Mangifera indica 'Namdokmai'	Gallic acid	169.05 / 124.99	169.05 / 79.18	0.546-0.556	2163.00-2352.00
	Quercetin	301.20 / 151.04	301.20 / 178.98	4.135-4.151	12088.00-13880.00
	Naringenin	272.94 / 153.05	272.94 / 146.97	4.660-4.672	745.00-795.00
	Kaempferol	285.15 / 239.11	285.15 / 186.99	4.740-4.750	106.00-118.00
	Isorhamnetin	315.09 / 300.00	315.09 / 150.97	4.820-4.832	1446.00-1680.00
Psidium guajava 'Kimju'	Gallic acid	169.05 / 124.99	169.05 / 79.18	0.546-0.556	45407.00-46080.00
	Quercetin	301.20 / 151.04	301.20 / 178.98	4.139-4.151	1354.00-1593.00

Note: The identifications of phenolics were based on the matching of 2 MS transitions (transition 1 and transition 2) from 2-3 MS transitions of authentic standards (as in Supplementary Table S2).

Supplementary Table S4:

Information on neurotoxicity and neuroprotective effect of the selected fruit extracts (*M. indica* 'Namdokmai', *M. indica* 'Keaw' and *P. guajava* 'Kimju')

Fruit extract	Incubating time (h)	Extract Concentration (μg/mL)				
		0	25	50	100	150
Mangifera indica 'Kaew'	24	95.56 ± 4.35	97.49 ± 1.92	93.37 ± 2.75	95.43 ± 3.79	91.53 ± 3.32
	48	98.04 ± 2.19	95.56 ± 4.60	98.05 ± 2.30	91.35 ± 1.60 *	86.06 ± 1.75 *
	72	101.60 ± 5.55	94.60 ± 2.88	94.18 ± 3.60	82.02 ± 1.78 *	78.66 ± 2.88 *
Mangifera indica 'Namdokmai'	24	98.22 ± 0.94	93.56 ± 2.46	94.35 ± 2.35	95.43 ± 3.79	95.42 ± 3.52
	48	98.04 ± 2.19	98.83 ± 3.82	94.78 ± 3.62	96.75 ± 3.15	94.01 ± 1.89
	72	99.25 ± 2.72	95.09 ± 2.23	92.92 ± 3.59	84.78 ± 3.98 *	79.93 ± 2.14 *
Psidium guajava 'Kimju'	24	97.87 ± 4.42	98.49 ± 1.65	96.67 ± 4.23	95.76 ± 4.35	91.35 ± 3.79
	48	98.97 ± 1.63	98.87 ± 3.52	98.10 ± 1.89	98.19 ± 1.76	86.05 ± 2.97 *
	72	101.00 ± 3.47	99.34 ± 2.71	92.38 ± 3.84	87.48 ± 2.44 *	73.64 ± 2.32 *

* Significance at $p < 0.05$ compared with untreated cells using two-way analysis of variance (ANOVA) followed by Tukey's multiple comparisons test.

Supplementary Table S5:

Information on neuroprotective effect of the selected fruit extracts (*M. indica* 'Namdokmai', *M. indica* 'Keaw' and *P. guajava* 'Kimju')

Conditions	Extract concentration (µg/mL)	% Cell viability			% Lactate dehydrogenase (LDH) release			% Relative light unit (RLU)		
		***M. indica* 'Kaew'**	***M. indica* 'Namdokmai'**	***P. guajava* 'Kimju'**	***M. indica* 'Kaew'**	***M. indica* 'Namdokmai'**	***P. guajava* 'Kimju'**	***M. indica* 'Kaew'**	***M. indica* 'Namdokmai'**	***P. guajava* 'Kimju'**
Untreated for H_2O_2 inducer			98.73 ± 0.93			13.10 ± 1.45			98.92 ± 2.50	
H_2O_2-treated	0		56.82 ± 1.97 #			54.83 ± 3.19 #			42.30 ± 2.66 #	
	25	62.72 ± 2.12 *	66.39 ± 1.78 **	60.68 ± 1.54 *	52.09 ± 2.28	46.48 ± 1.36 ***	53.41 ± 3.82	46.64 ± 1.60 *	56.90 ± 1.44 *	50.83 ± 3.19 *
	50	64.76 ± 1.33 ***	69.27 ± 2.22 ***	65.64 ± 1.55 ***	49.78 ± 1.69 *	41.16 ± 1.66 ***	47.97 ± 1.18 ***	54.67 ± 2.11 ***	62.64 ± 2.82 ***	59.52 ± 1.31 ***
	100	65.88 ± 1.77 ***	73.43 ± 1.90 ***	66.63 ± 1.70 ***	44.93 ± 1.69 ***	34.27 ± 3.57 ***	45.39 ± 1.30 ***	64.50 ± 1.83 ***	73.57 ± 1.59 ***	68.68 ± 1.09 ***
	150	72.57 ± 1.91 ***	81.90 ± 2.20 ***	75.54 ± 1.49 ***	36.18 ± 2.18 ***	15.56 ± 0.67 ***	35.85 ± 2.28 ***	71.98 ± 1.67 ***	86.47 ± 2.18 ***	76.61 ± 2.27 ***
Untreated for $A\beta_{1\text{-}42}$ inducer			99.50 ± 1.91			13.22 ± 1.39			97.90 ± 2.20	
$A\beta_{1\text{-}42}$ treated	0		50.92 ± 3.68 #			52.83 ± 2.35 #			49.00 ± 3.41 #	
	25	51.37 ± 2.41	46.48 ± 1.36	53.92 ± 2.54	51.37 ± 2.41	46.48 ± 1.36 **	53.92 ± 2.54	53.23 ± 3.97	57.20 ± 1.61 **	51.65 ± 5.30
	50	57.43 ± 3.44 **	59.47 ± 3.19 **	60.09 ± 4.42 ***	47.28 ± 2.54 *	41.16 ± 1.66 ***	45.82 ± 2.07 ***	59.33 ± 1.49 ***	63.28 ± 2.86 ***	60.95 ± 1.83 ***
	100	63.26 ± 1.05 ***	71.94 ± 3.35 ***	69.30 ± 1.07 ***	39.55 ± 2.22 ***	27.83 ± 1.66 ***	37.83 ± 2.07 ***	64.09 ± 2.05 ***	72.35 ± 2.68 ***	66.91 ± 1.74 ***
	150	70.89 ± 2.30 ***	82.62 ± 3.37 ***	76.28 ± 2.40 ***	37.19 ± 1.84 ***	25.00 ± 3.16 ***	33.48 ± 3.80 ***	71.98 ± 1.67 ***	82.48 ± 2.70 ***	75.33 ± 2.54 ***

Significance at $p < 0.05$ compared to the untreated control, * significance at $p < 0.05$, ** significance at $p = 0.01$ and *** significance at $p = 0.001$ compared with either H_2O_2 or $A\beta_{1\text{-}42}$-treated cells using one-way analysis of variance (ANOVA) followed by Tukey's multiple comparisons test.

Supplementary Table S6:

Images of fruit samples including *Ananas comosus* ‘Phulae’, *Ananas comosus* ‘Pattavia’, *Carica papaya* ‘Khaekdum’, *Carica papaya* ‘Khaeknuan’, *Durio zibethinus* ‘Monthong’, *Durio zibethinus* ‘Chanee’, *Psidium guajava* ‘Kimju’, *Psidium guajava* ‘Keenok’, *Mangifera indica* ‘Kaew’, and *Mangifera indica* ‘Namdokmai’.

Fruits	Physical appearance	
Ananas comosus	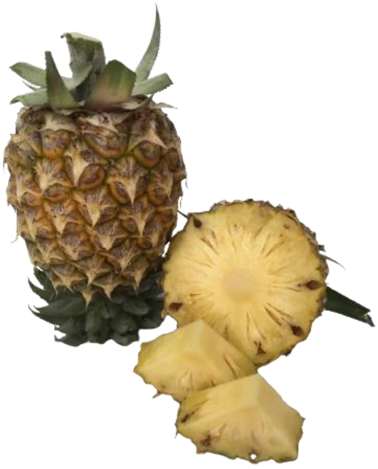 ‘Phulae’	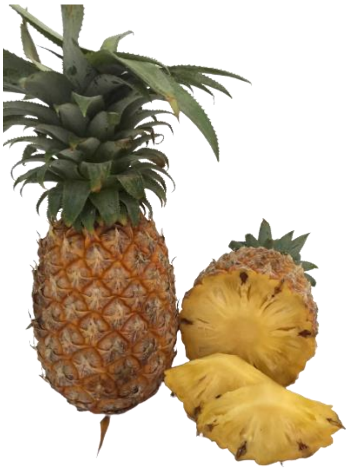 ‘Pattavia’
Carica papaya	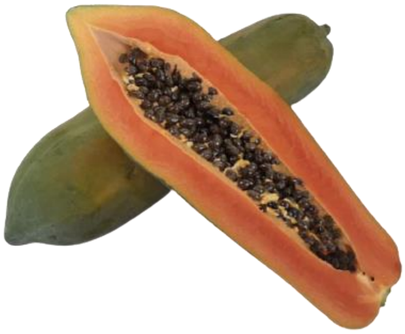 ‘Khaekdum’	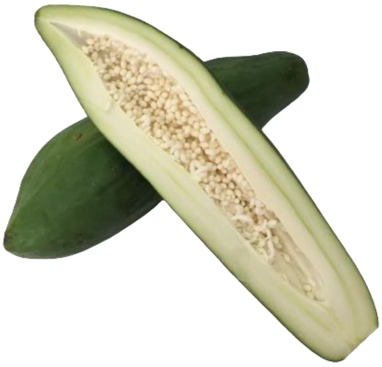 ‘Khaeknuan’
Durio zibethinus	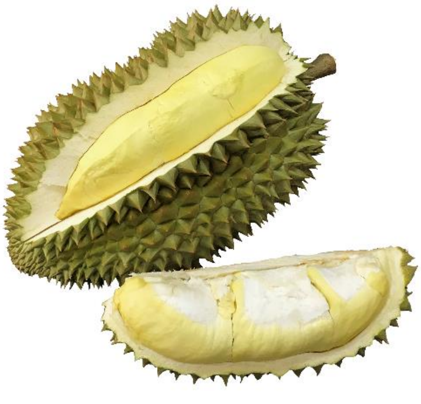 ‘Monthong’	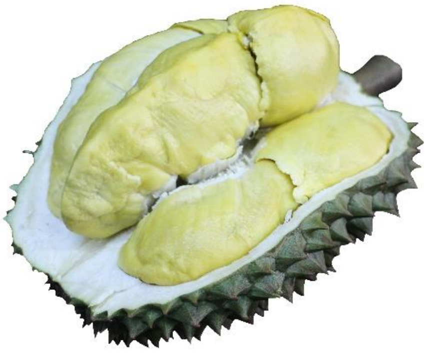 ‘Chanee’

Supplementary Table S6 (Cont.):

Images of fruit samples including *Ananas comosus* 'Phulae', *Ananas comosus* 'Pattavia', *Carica papaya* 'Khaekdum', *Carica papaya* 'Khaeknuan', *Durio zibethinus* 'Monthong', *Durio zibethinus* 'Chanee', *Psidium guajava* 'Kimju', *Psidium guajava* 'Keenok', *Mangifera indica* 'Kaew', and *Mangifera indica* 'Namdokmai'.

**Fruits**	**Physical appearance**	
Psidium guajava	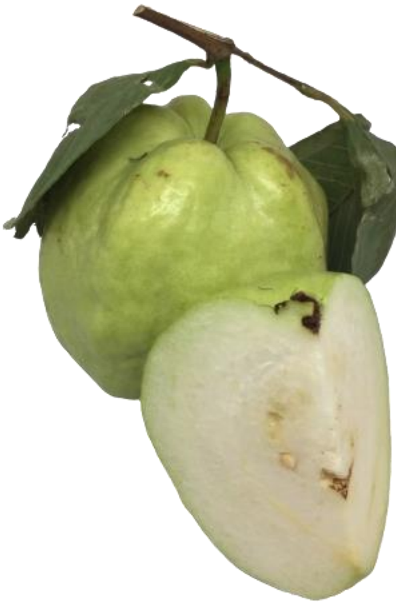<br>'Kimju'	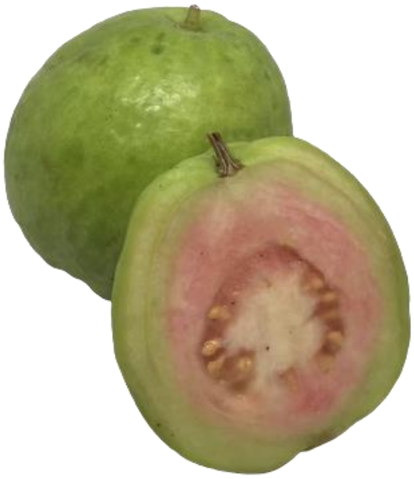<br>'Keenok'
Mangifera indica	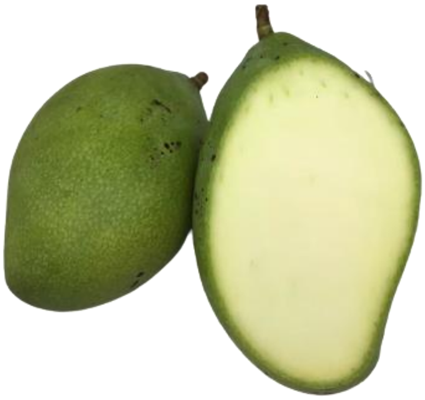<br>'Kaew'	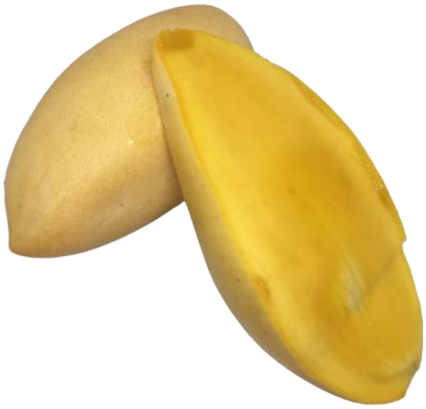<br>'Namdokmai'

Supplementary Figure S1:

The liquid chromatography–electrospray ionization tandem mass spectrometry (LC–ESI-MS/MS) chromatograms of 24 phenolic standards.

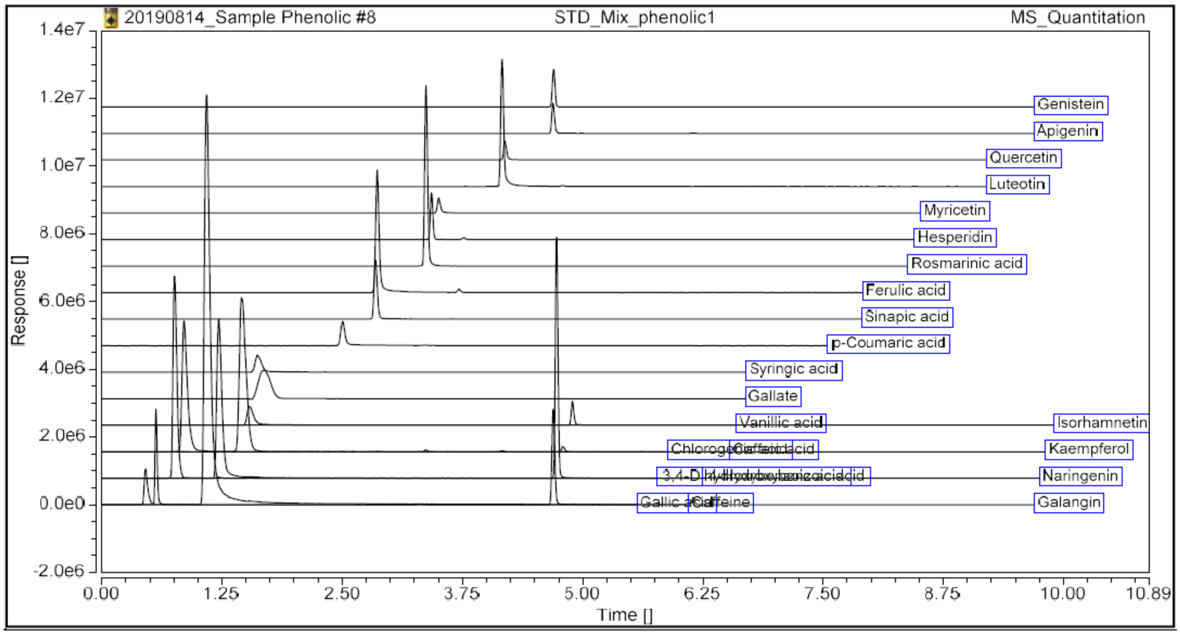

Supplementary Figure S2:

The liquid chromatography–electrospray ionization tandem mass spectrometry (LC–ESI-MS/MS) chromatograms of the selected fruit samples including (**a**) *Mangifera indica* ‘Kaew’, (**b**) *Mangifera indica* ‘Namdokmai’, and (**c**) *Psidium guajava* ‘Kimju’.

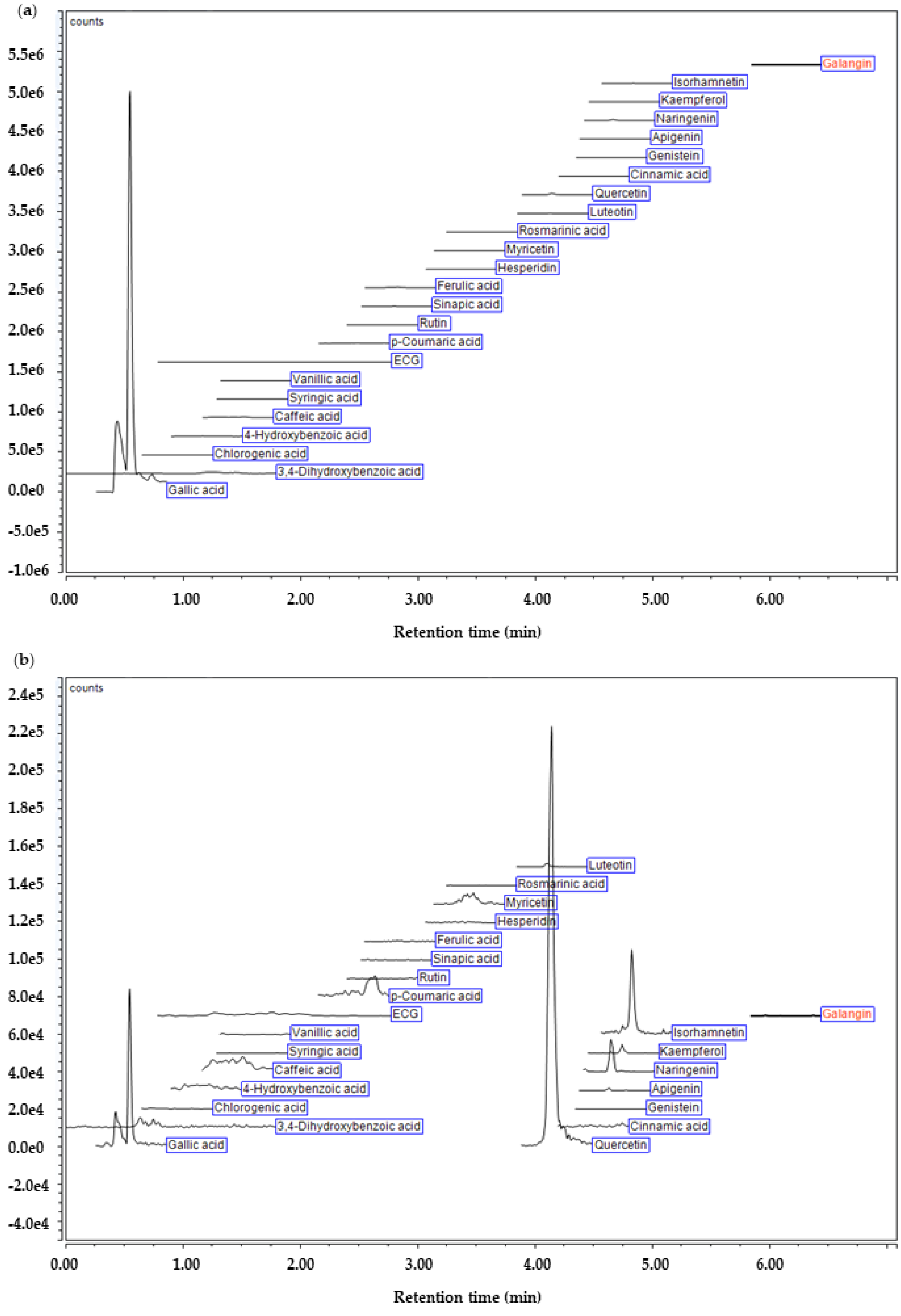

Supplementary Figure S2 (cont.):

The liquid chromatography–electrospray ionization tandem mass spectrometry (LC–ESI-MS/MS) chromatograms of the selected fruit samples including (**a**) *Mangifera indica* 'Kaew', (**b**) *Mangifera indica* 'Namdokmai', and (**c**) *Psidium guajava* 'Kimju'.

(**c**)

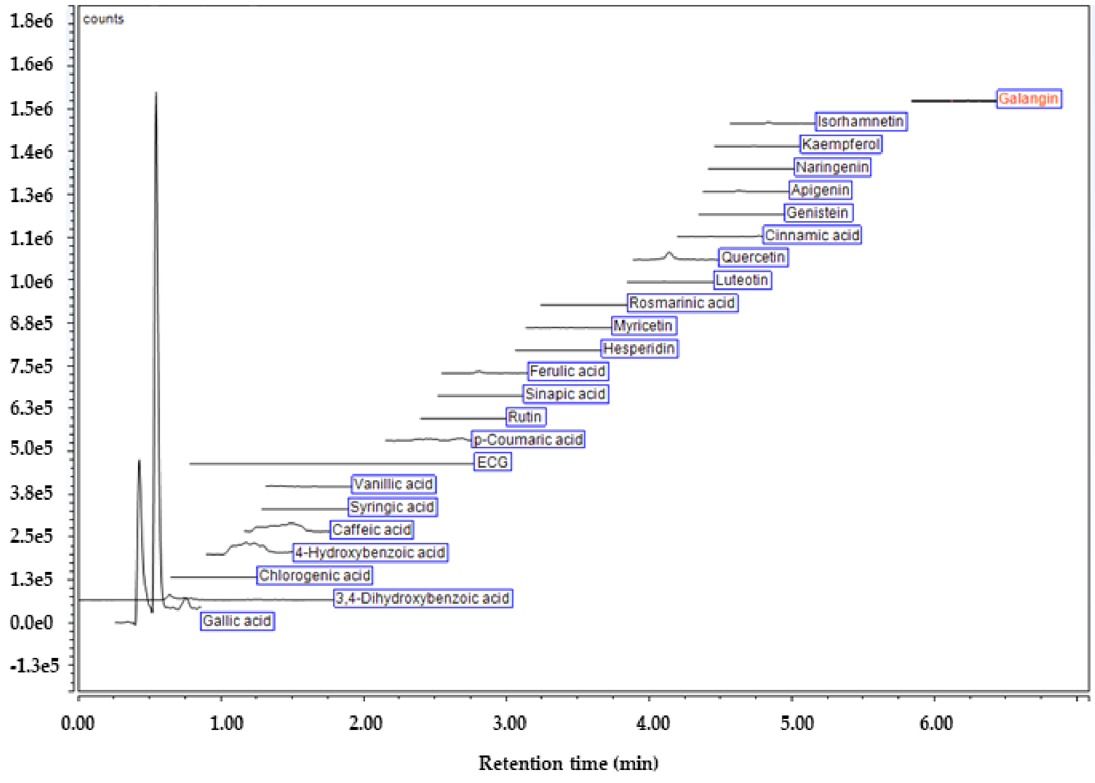

Supplement: Supplementary file 1 [file pharmaceuticals-15-00591-s001.zip › pharmaceuticals-1640214-supplementary.pdf]
